# Phyllosphere-associated microbiota in built environment: Do they have the potential to antagonize human pathogens?

**DOI:** 10.1016/j.jare.2022.02.003

**Published:** 2022-02-12

**Authors:** Wisnu Adi Wicaksono, Tamara Reisenhofer-Graber, Sabine Erschen, Peter Kusstatscher, Christian Berg, Robert Krause, Tomislav Cernava, Gabriele Berg

**Affiliations:** a Institute of Environmental Biotechnology, Graz University of Technology, Graz, Austria; b Institute of Plant Sciences, Karl-Franzens-University, Graz, Austria; c Department of Internal Medicine, Medical University of Graz, Graz, Austria; d BioTechMed Graz, Inter-university Cooperation Platform, Graz, Austria; e Leibniz Institute for Agricultural Engineering and Bioeconomy Potsdam, Potsdam, Germany; f Institute for Biochemistry and Biology, University of Potsdam, Potsdam, Germany

**Keywords:** Human health, Indoor plant microbiome, Indoor microbiome, Built environment, DNA, deoxyribonucleic acid, PCR, polymerase chain reaction, rRNA, ribosomal ribonucleic acid, ITS, intergenic transcript spacer, ASV, amplicon sequence variant, ARG, antibiotic resistance gene, MAG, metagenome assembled genome, NMDS, non-metric multidimensional scaling, PERMANOVA, permutational analyses of variance, Nutrient Broth II agar, NA, MHA, Mueller Hinton agar, COG, Clusters of Orthologous Groups of *proteins*, MLS, macrolide-lincosamide-stretogramin

## Abstract

**Introduction:**

The plant microbiota is known to protect its host against invasion by plant pathogens. Recent studies have indicated that the microbiota of indoor plants is transmitted to the local built environment where it might fulfill yet unexplored functions. A better understanding of the interplay of such microbial communities with human pathogens might provide novel cues related to natural inhibition of them.

**Objective:**

We studied the plant microbiota of two model indoor plants, *Musa acuminata* and *Chlorophytum comosum,* and their effect on human pathogens. The main objective was to identify mechanisms by which the microbiota of indoor plants inhibits human-pathogenic bacteria.

**Methods:**

Microbial communities and functioning were investigated using a comprehensive set of experiments and methods combining amplicon and shotgun metagenomic analyses with results from interaction assays.

**Results:**

A diverse microbial community was found to be present on *Musa* and *Chlorophytum* grown in different indoor environments; the datasets comprised 1066 bacterial, 1261 fungal, and 358 archaeal ASVs. Bacterial communities were specific for each plant species, whereas fungal and archaeal communities were primarily shaped by the built environment. *Sphingomonas* and *Bacillus* were found to be prevalent components of a ubiquitous core microbiome in the two model plants; they are well-known for antagonistic activity towards plant pathogens. Interaction assays indicated that they can also antagonize opportunistic human pathogens. Moreover, the native plant microbiomes harbored a broad spectrum of biosynthetic gene clusters, and in parallel, a variety of antimicrobial resistance genes. By conducting comparative metagenomic analyses between plants and abiotic surfaces, we found that the phyllosphere microbiota harbors features that are clearly distinguishable from the surrounding abiotic surfaces.

**Conclusions:**

Naturally occurring phyllosphere bacteria can potentially act as a protective shield against opportunistic human pathogens. This knowledge and the underlying mechanisms can provide an important basis to establish a healthy microbiome in built environments.

## Highlights

• Fungal and archaeal communities of indoor plants are primarily shaped by the built environment.
• Bacterial communities are host-specific irrespective of the environment.
• Core bacterial members can potentially act as a protective shield against human pathogens.
• The plant microbiota harbours various intrinsic functions to counteract establishment of pathogens.
• The production of bioactive compounds and a versatile resistome play important roles in the defence.

## Graphical abstract

Schematic visualization of the discovered implications of indoor-plant-associated bacteria as a defense line against opportunistic human pathogens. Text in ellipses: All relevant functions of the bacterial community confirmed in this study that serve to shield off pathogens. Text in rectangles: Methods and techniques that were implemented to analyze the respective functions.

**Figure:**
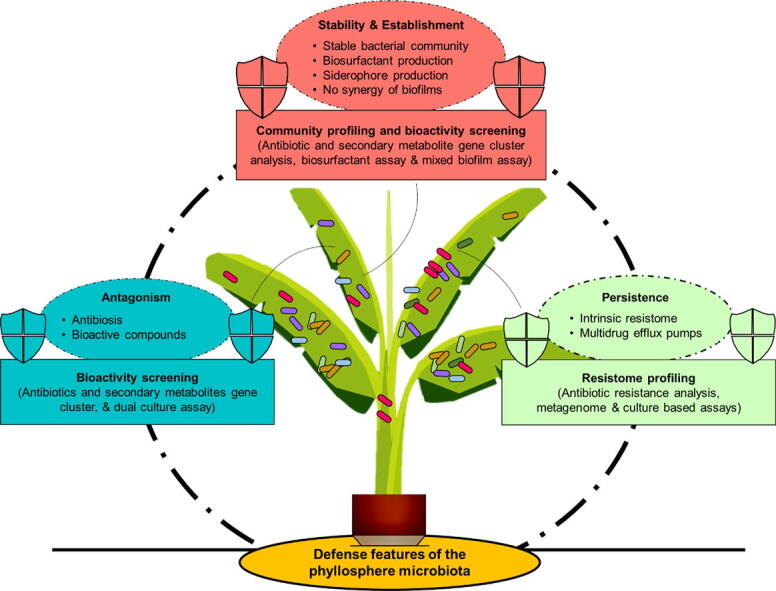

## Introduction

Over the last few years, evidence has been accumulating that there is a link between the plant and the human microbiota that affects human health [1], [2]. Intake of plant-associated microbes through food consumption is considered the main source for exposure of the human microbiome to natural microbiomes [3]. Moreover, the direct contact with plants was previously shown to affect the human skin microbiota [4]. Recent studies revealed the importance of indoor plant-associated microbes [5], [6] and their impact on microbial diversity in the built environment [5], [7]. This can be significant in terms of human health [8], as extensive cleaning regimes can result in a loss of microbial diversity and increased occurrence of antimicrobial resistance [9]. Nevertheless, targeted studies related to beneficial effects of indoor plants on human health are still scarce.

Plants naturally harbor numerous useful microorganisms that act as antagonists against plant pathogens or promote growth; but also harbor potential human pathogens, e.g. *Staphylococcus aureus*, *Streptococcus pneumoniae*, *Stenotrophomonas maltophilia*, *Escherichia coli,* and *Salmonella enterica* [10], [11], [12]. Known modes of action to suppress plant pathogens include colonization and biofilm formation by beneficial microorganisms as well as pathogen suppression by antibiosis, competition and lysis [13], [14], [15], [16]. The frequent presence of antibiotics, biosurfactans, and antimicrobial volatiles requires a protective mechanism of the native plant microbiota commonly known as the “resistome” [17], [18], [19]. We hypothesized that the phyllosphere microbiota of plants grown in the built environment can suppress opportunistic human pathogens by employing its natural repertoire of defense mechanisms which includes their intrinsic resistome. Until now, the role of the resistome has received little attention, although it is considered to be an important component of the natural defense against biotic stresses [18], [19].

To gain deeper insights into the microbiome of indoor plants and evaluate their potential for suppressing human pathogenic bacteria, we performed a series of experiments by combining meta-omics analyses and cultivation assays. We explored the phyllosphere microbiome of two representative indoor plants species *Musa acuminata* Colla (dwarf banana) and *Chlorophytum comosum* (Thunb.) Jacques (spider plant) from built environments with varying degrees of human influence. These plants are common indoor plants worldwide. In addition, *C. comosum* and *M. acuminata* have already been shown to have a positive impact on indoor air quality by reducing air pollutants such as formaldehyde, xylene, and toluene [20], [21], [22]. Moreover, *C. comosum* was shown to affect microbial diversity and abundance in its surrounding environment [5]. To comprehensively assess the phyllosphere microbiome of the model plants, we designed a sampling strategy that included public houses and greenhouses of a botanical garden as well as commercial stores where these houseplants can be purchased. We addressed the following general questions: (i) Are there differences in the indoor-plant-associated microbiomes? (ii) Is there a specific core microbiome shared by all analyzed indoor plants? (iii) Can the commonly present phyllosphere microbiota of the studied plants counteract opportunistic human pathogens? Specific interactions were assessed using dual-culture and mixed-biofilm assays against opportunistic human pathogens and by screening for possible modes of action in metagenomic libraries. Moreover, to fill the knowledge gap regarding potentially beneficial functions of the microbiota and to analyze their natural resistomes, a combination of cultivation assays and complementary metagenomic analyses was performed, addressing the following specific question: (iv) How is the phyllosphere resistome structured and how does it compare to other built environment surfaces? Overall, this study provides important insights into the ecological function and potential beneficial effects of the indoor plant microbiota on human health.

## Material and methods

### Sampling and processing of plant material

Leaf samples of *Chlorophytum comosum* (Thunb.) Jacques and *Musa acuminata* Colla were collected between July and August 2019 from multiple locations within Styria (Austria). A total of six indoor built environments consisting of the Graz Botanical Garden (n = 1), private homes (n = 3), and commercial stores (n = 2) were chosen as the sampling sites (Table S1, Fig. S1). The Graz Botanical Garden complex has four different closed areas that are mainly composed of glass panels without direct exposure to the outdoor environment. We therefore assumed that the effects of external factors known to affect the plant phyllosphere, i.e., airflow, humidity, and temperature, would be lowest at this site. Plants from the Graz Botanical Garden were also grown indoor for extended time periods under controlled conditions (*i.e*. temperature and humidity). In contrast, the other built environments were not controlled for these parameters. At least three healthy leaves from each individual plant of *C. comosum* were chosen randomly and pooled for DNA extraction described below, whereas one leaf from *M. acuminata* was sampled by using sterile gloves and instruments. A total of 37 plant samples were collected from six indoor built environments (Table S1). The leaf samples were kept in 25 × 32 cm bags (ARO freezer bags; Düsseldorf, Germany) and kept cool at 4 °C until laboratory processing. The leaf samples were processed using a stomacher (BagMixer; St. Nom, France) and a Transsonic Digital T910 DH sonicator (Elma; Singen, Germany) within 24 h after collection as described previously [6] to obtain leaf epiphyte samples. The leaf wash-offs were then transferred to a 50-ml Sarstedt tube and used for total community DNA extraction and cultivation of phyllosphere bacteria as described below.

### Preparation of amplicon and shotgun sequencing-based metagenomic datasets

*DNA extraction*

The leaf wash-offs were centrifuged (Sorvall RC-5B Refrigerated Superspeed Centrifuge; DuPont Instruments, USA) at 6000 g for 20 min to pellet cells. The pellets were then transferred to 2-ml sterile Eppendorf tubes and were further centrifuged at 16 000 g for 20 min. DNA extractions were performed using the FastDNA™ SPIN Kit for Soil (MP Biomedicals, Santa Ana, CA, United States) following the manufacturer’s instructions.

### Amplicon sequencing of total microbial community DNA extracts

Extracted DNA was subjected to PCR-based amplifications to target the bacterial, fungal and archaeal community. The set of bacteria-specific primers 515f/926r [23] that also contained barcode sequences for multiplexing was used to amplify the V4-V5 region of bacterial 16S rRNA gene. Another set of fungi-specific primers ITS1f/ITS2 [24] was used to amplify the ITS1 region of fungal intergenic transcript spacer (ITS) gene and the specific primer pair 349f/519r was used to amplify 16S rRNA gene fragments of the archaeal community [25], [26]. PCR products were further purified using the Wizard SV Gel and PCR Clean-Up System (Promega, Madison, WI), pooled in equimolar concentrations and sequenced using the Illumina MiSeq platform (2 × 300 bp; paired-end sequencing) at the commercial provider Genewiz (Leipzig, Germany). Amplicon sequences were deposited at the European Nucleotide Archive (ENA) under the project number PRJEB38711.

### Shotgun metagenomics sequencing

For this approach, samples from the botanical garden were chosen because the plants were grown under the most controlled conditions at this location. Six shotgun libraries (1 µg DNA per library) consisted of the two different plant species with three biological replicates each. The subsequent DNA library preparation and shotgun metagenomic sequencing using the Illumina HiSeq platform (2 × 150 bp; paired-end sequencing) was performed by the commercial provider Genewiz (Leipzig, Germany). Shotgun metagenome sequences were deposited at the European Nucleotide Archive (ENA) under the project number PRJEB38967. We further retrieved publicly available shotgun metagenomes of abiotic surfaces that are representative for (i) naturally unrestricted buildings and (ii) controlled built environments from a previous study [9]. To study the specificity of the indoor plant phyllosphere microbiome, metagenomes of C. comosum and *M. acuminata* were compared to metagenomes obtained from abiotic surfaces with respect to microbial community and antibiotic resistance gene composition as described below.

### Bioinformatic analyses

For amplicon sequencing, paired-end sequences were quality checked and demultiplexed using cutadapt, [27]. Due to high occurrence of low-quality reads on the reverse pairs, only the forward reads were used for bacterial community analysis. Quality filtering, trimming, denoising, merging and chimera removal of paired end sequences were performed using the DADA2 algorithm [28] through the open-source QIIME2 [29]. The resulting amplicon sequencing variants (ASVs) were taxonomically assigned using VSEARCH classifier [30] against the Silva ribosomal RNA gene database v128 [31] for the bacterial and archaeal datasets and UNITE + INSD (v6_sh_97) for the fungal dataset [32]. Plant-derived sequences such as chloroplasts and mitochondria were removed prior further statistical analyses. One sample had to be removed from the bacterial amplicon library due to its low number of reads after quality filtering. The final datasets contained 1.74 × 10^5^ bacterial reads, 2.92 × 10^5^ fungal reads, and 6.41 × 10^5^ archaeal reads. These reads were assigned to 1066 bacterial, 1261 fungal and 358 archaeal ASVs (Table S2).

For shotgun metagenomic sequencing, high quality reads were used as input for taxonomic profiling using the metagenomic classifier Kraken2 [33] and Bracken [34]. The filtered reads were then assembled using Megahit [35]. Prodigal was used to predict coding DNA sequences of the assembled contigs [36]. To create a non-redundant gene catalogue, CD-HIT-EST was used to cluster predicted protein-coding gene sequences at 95% nucleotide identity [37]. Representative gene sequences were then annotated using the blast algorithm in DIAMOND against the eggNOG database v5.0 [38]. The assembled contigs were further aligned to the manually curated antibiotic resistance gene database (deepARG, [39]) for antibiotic resistance gene (ARGs) profiling. To minimize the risk of false positives, contigs were defined as ARG-like contigs at a defined cut-off with an E-value of 10^-10^ and similarity of 50% as previously described [18]. The Burrows-Wheeler Aligner [40] was used to align individual metagenomic dataset to the annotated contigs and to count the number of reads, respectively. Only reads mapped to contigs with eggNOG and deepARG features were used for further analysis. Antibiotic resistance gene carriers were identified by uploading the ARG-like contigs to the Kaiju web server [41]. Biosynthetic gene clusters were predicted from the indoor plant phyllosphere metagenome using antiSMASH [42] with assembled contigs and metagenome assembled genomes (MAGs) as input data and the prodigal tool for gene prediction [36] as previously described [43]. Before the analyses, all MAGs were generated by combining various binning methods, i.e. Maxbin2, MetaBAT2, and CONCOCT v1.1.0 [44], [45], [46] and the assembled metagenomic contigs as input. Subsequently, the MAGs were dereplicated using DASTool v1.1.1 [47]. The quality of metagenome assembled genomes (completeness and contamination) was assessed using checkM v1.0.13 [48]. Taxonomical information of the MAGs was obtained using GTDB-Tk v1.4.1 [49].

### Statistical analysis

Deepening microbial community analyses were performed in R v3.3.1 [50] through the Rstudio IDE (using http://www.rstudio.com/) with phyloseq packages [51]. For alpha and beta diversity analyses, the number of sequences was normalized by randomly selecting an equal number of sequences according to the lowest common number of sequences. The Kruskal-Wallis test was used to evaluate effects of plant species and sampling site on the microbial richness according to the Chao1 index. Bray-Curtis dissimilarity matrix distances were generated and subjected to permutational analyses of variance (PERMANOVA, 999 permutations) along with the adonis test to evaluate effects of plant species and sampling site on microbial richness on the microbial community structure. The same analysis was also used to evaluate the effect of the sample type (plant surfaces and abiotic surfaces) on bacterial and ARG composition. A non-metric multidimensional scaling (NMDS) plot was generated to ordinate the normalized Bray-Curtis dissimilarity matrix. For analysing ARG compositions, the Bray-Curtis dissimilarity matrix was implemented to generate hierarchical clustering [51]. Correlation network analysis (Spearman rank correlation) at genus level was performed using MicrobiomeAnalyst web browser [52].

### Functional characterization of cultivable phyllosphere bacteria

Isolation of bacteria from the plant phyllosphere

For the isolation of bacteria, samples from the botanical garden were chosen to limit the number of isolates and allow comparisons with microbial functioning derived from the metagenomic datasets. A total of 100 μl of the leaf wash-offs was serially diluted 10-fold and plated on both Reasoner's 2A (R2A) (Carl Roth GmbH + Co. KG; Karlsruhe, Germany) and Nutrient Broth II agar (NA) media (SIFIN, Berlin, Germany) in triplicates and incubated at 25 °C for four days. Single colonies were picked and sub-cultured on NA to ensure purity of the isolates. Representative bacterial colonies were selected based on differences in colony morphology (shape and color) from each dilution in order to increase the number of unique isolates. Colonies were then transferred to 96-well plates with Nutrient Broth II and 30 % glycerol for long-term storage and the plates were kept at −70 °C at the Institute of Environmental Biotechnology, Graz University of Technology, Graz, Austria. In total, 389 isolates (n = 264 from *C. comosum* and n = 125 from M. acuminata) were collected and used for functionality assays. All phyllosphere isolates were tested in three replicates to increase the reliability of the screening assays that are described below.

### Identification of culturable bacteria based on 16S rRNA gene sequencing

Bacterial isolates were identified based on the sequence of their 16S rRNA gene fragments using the primer set 27F and 1492R. Genomic DNA was extracted using the MasterPure™ Complete DNA and RNA Purification Kit (Epicentre, Madison, WI, USA) according to manufacturer’s instructions with an additional mechanical cell disruption step with glass beads in a FastPrep Instrument (MP Biomedicals, Santa Ana, CA, USA; 30 s, 6.5 m s^−1^). Polymerase chain reactions (PCRs) were performed with a Whatman Biometra® Tpersonal thermocycler (Biometra 141 GmbH, Göttingen, Germany) as described previously in [6]. The amplified 16S rRNA genes were Sanger sequenced at the commercial sequencing provider LGC Genomics (Berlin, Germany). After manual quality filtering to remove ambiguous sequences using BioEdit [53], the sequences were compared against database entries using the Basic Local Alignment Search Tool (BLAST and the GenBank database (http://www.ncbi.nlm.nih.gov). Subsequently, the sequences were aligned using MUSCLE [54], and the distance matrices for the phylogenetic tree were calculated with the maximum-likelihood algorithm in MEGA X (Molecular Evolutionary Genetic Analysis) [55]. The phylogenetic tree was visualized using the interactive tree of life software (iTOL, [56]).

### Antagonistic activity against opportunistic human pathogens

Phyllosphere bacteria were spotted (10 μl) on NA plates pre-inoculated with human opportunistic pathogens including *Acinetobacter baumanii*, *Enterococcus faecium*, *Escherichia coli*, *Staphylococcus haemolyticus*, *Stenotrophomonas maltophilia* and *Pseudomonas aeruginosa*. *A. baumanii*, *E. faecium*, *E. coli,* and *P. aeruginosa* isolates were obtained from culture collection of Department of Internal Medicine, Medical University of Graz. The *S. haemolyticus* strain 48-6 was previously isolated from a hospital setting [57] while *S. maltophilia* was previously isolated from eye care solution and shown to be closely related to a clinical strain, *S. maltophilia* K279a [58]. The latter two isolates are part of the microbial culture collection of the Institute of Environmental Biotechnology (Graz University of Technology). Inhibition zones on the agar surface were assessed after 4 days of incubation at 25 °C. All isolates that produced visible inhibition zones were classified as antagonists of the model pathogens.

### Biofilm formation and biofilm co-culture assay

Biofilm production is one of the mechanisms by which opportunistic pathogens counteract antimicrobials and cleaning regimes to thrive under typical hospital settings [59], [60], [61], [62]. We therefore further investigated the synergism between phyllosphere-associated bacteria and our collection of opportunistic human pathogens by implementing a biofilm co-culture assay. The biofilm formation assay was performed in 96-well microtiter plates made of polystyrene as previously described [63]. Biofilm quantifications were done with a crystal violet (CV) assay and OD_595_ measurements for each well using the Tecan microplate reader Infinite® 200 PRO (Tecan Austria GmbH, Grödig, Austria). The biofilm formation was examined for each phyllosphere isolate, for single pathogens as well as for co-cultures that contained a phyllosphere bacterium and a pathogen. The pathogens implemented in the assay were *E. faecium, S. maltophilia* and *P. aeruginosa*. The interactions in the mixed biofilms (MBA) were defined as synergistic if the measured absorbance from the mixed biofilm was greater than that of the best single strain biofilm (BSA) producer of that mixture. In contrast a non-synergistic effect shows a lower absorbance of the mixture compared to the single strains, i.e., (Abs_595_ MBA - Standard error) > (Abs_59__5_ BSA + Standard error) = Synergy, while (Abs_595_ MBA + Standard error) < (Abs_595_ BSA - Standard error) = No synergy [64].

### Screening for biosurfactant-producing bacteria

A qualitative screening of biosurfactant producing bacteria was performed using the drop-collapsing assay as previously described [65]. Briefly, the 96 wells of the lid of a 96-well microtiter plate were covered with 2 μl mineral oil and incubated for 2 h to equilibrate. Afterwards, 5 μl of a liquid single cell culture of the phyllosphere bacteria were added to the mineral oil and the result was evaluated after 1 min. A drop that remained beaded was determined as negative while collapsed drops were determined as positive.

### Antibiotic resistance profiling

Bacterial isolates were screened against 11 different antibiotics (Table S3). Agar plate-based assays were performed as previously described [66], [67]. In brief, Mueller Hinton agar (MHA) plates containing a specific antibiotic at 20 µg/ml were inoculated with approximately 3 µl bacterial culture using a multipoint inoculator and were then incubated at 25 °C. MHA plates without antibiotic supplementation were used as controls. The inoculated plates were observed daily for three days. The experiments were performed in triplicates. Bacteria that showed visible growth after 4 days incubation on agar plates containing antibiotics were classified as resistant bacteria.

## Results

### Compositions of bacterial and fungal communities associated with indoor plants were influenced by plant species and the built environment

**Fig. 1:**
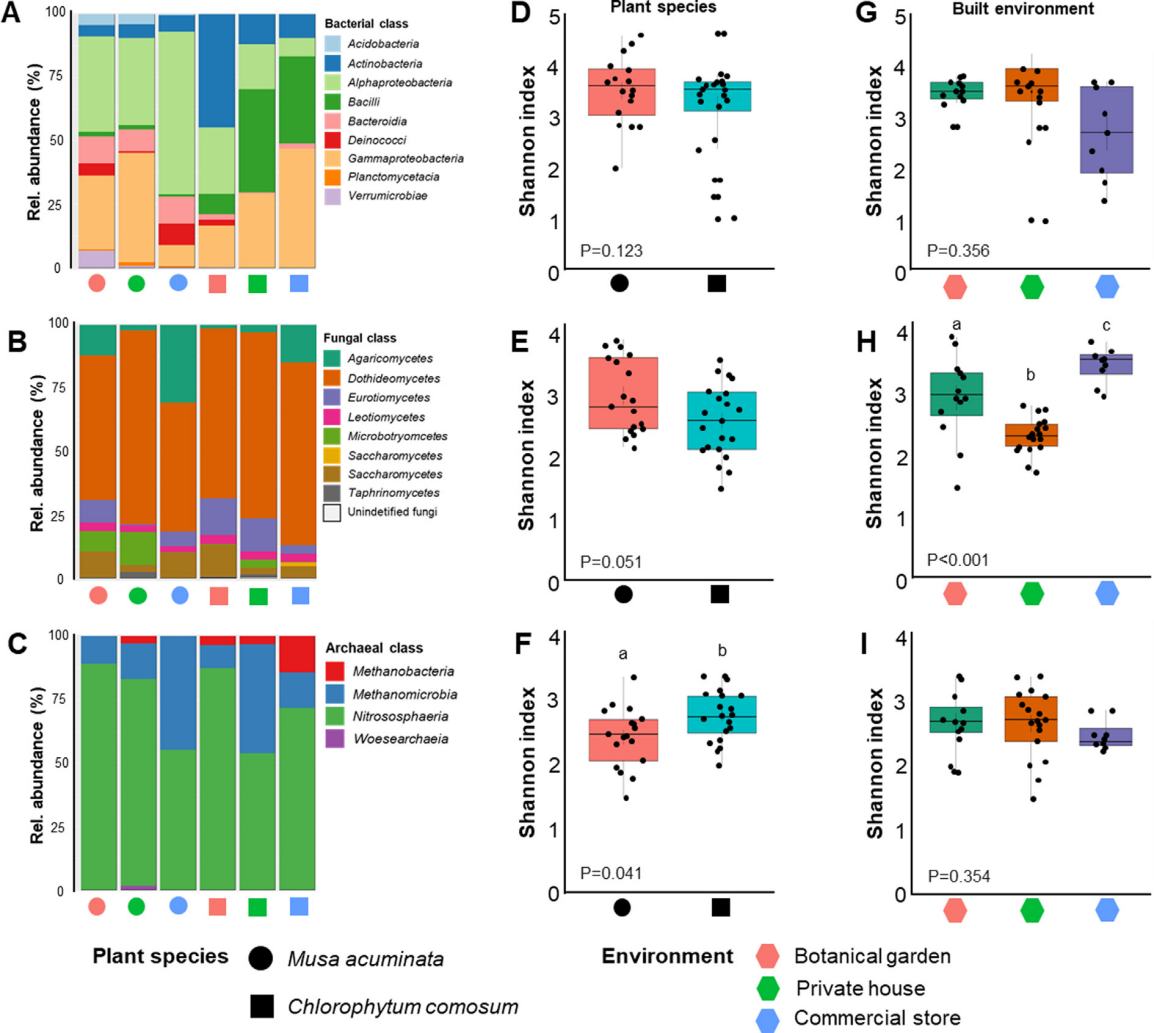
**Microbial community composition and diversity assessments.** The relative abundance of the top 100 bacterial (A), fungal (B) and archaeal (C) taxa was visualized on class level. The microbial diversity was compared between samples from the two plants *Musa acuminata* and *Chlorophytum comosum* (D-F) and the three studied environments (G- H). In order to compare bacterial (D and G), fungal (E, H) and archaeal (F and I) alpha diversity, the Shannon index was calculated for each sample group.

In the first step, general information was obtained on the composition of microbial communities detected in the phyllosphere of plants from different built environments. Plant species as well as built environments were shown to affect the structure of phyllosphere microbial communities. *Alphaproteobacteria*, *Gammaproteobacteria* and *Bacteroidia* were the predominant bacterial classes in *M. acuminata* and together accounted for 78% of the total reads. In contrast, *Bacilli*, *Gammaproteobacteria* and *Actinobacteria* were the dominant classes in *C. comosum* and accounted for 70.7% of the total reads (Fig. 1A). When the community of both plant species was assessed in more detail, specific differences in proportions of bacterial groups were evident. *Alphaproteobacteria* occurrence was relatively higher in *M. acuminata* obtained from commercial stores (54.6%) compared to the other sampling sites (36.1–38.4%) whereas *Gammaproteobacteria* showed the opposite trend. A higher abundance of *Actinobacteria* was observed in *C. comosum* from the botanical garden (39.6%) compared to the other sampling sites (12.1–13.2%), whereas *Gammaproteobacteria* and *Bacilli* showed the opposite trend. *Dothideomycetes* was identified as the dominant fungal class which accounted for 64.2% of the total reads (Fig. 1B). The abundance of *Agaricomycetes* was relatively higher in *M. acuminata* and *C. comosum* from commercial stores (35.7 and 17.4%, respectively) compared to other sites (1.8–15.4%). *Nitrososphaeria* and *Methanomicrobia* dominated the archaeal community and represented 94.6% of the total reads (Fig. 1C). We found a higher proportion of the archaeal class *Methanomicrobia* in *M. acuminata* from commercial stores and *C. comosum* obtained from private houses when compared to the other samples.

With respect to the plant species, a higher bacterial diversity was observed for *M. acuminata* (H = 3.4) compared to *C. comosum* (H = 3.1) (Fig. 1D). When different environments were assessed, samples from private houses and the botanical garden had a relatively higher bacterial alpha diversity (H = 3.4) compared to samples from commercial stores (H = 2.6; Fig. 1G). However, significance analyses based on the Kruskal-Wallis test indicated that plant species and environment did not influence bacterial diversity (P = 0.356 and P = 0.123, respectively; Fig. 1D and 1G). Similar to the bacterial fraction, a higher fungal diversity was detected in *M. acuminata* (H = 2.9) compared to *C. comosum* (H = 2.5), but the differences were not statistically significant at the 95% confidence level (Fig. 1E). In contrast, the environment was shown to influence fungal diversity (P < 0.001; Fig. 1H). Samples from commercial stores were associated with higher fungal diversity (H = 3.4) compared to samples from the botanical garden (H = 2.9) and private houses (H = 2.3). Moreover, a higher archaeal diversity was observed in *C. comosum* (H = 2.7) compared to *M. acuminata* (H = 2.4, P = 0.041; Fig. 1F). The environment, however, did not influence archaeal diversity (P = 0.354, Fig. 1I).

### A core microbiome in the phyllosphere of indoor plants across built environments was identified

*Bacillus*, *Sphingomonas, Microbacterium,* and *Burkholderia* were identified as core members within the bacterial community in *C. comosum* that were present in at least 75% of the total samples (Fig. S2). *Sphingomonas* and *Bacilllus* were also identified as a core phyllosphere members of *M. acuminata*. Other taxa such as *Rhizobium*, *Methylobacterium*, *Pseudomonas,* and *Deinococcus* occurred in at least 90% of the *M. acuminata* samples. The core fungal community was composed of not further classifiable members of *Capnoidales*, *Alternaria* and *Mycosphaerella* in both plant species. In the archaeal community, one unidentified member of the family *Nitrososphaeraceae* and one representative that was only assignable to *Archaea*, as well as a member of the genus *Methonosarcina* were identified as members of the core community and found in at least 75% of the total samples from both plant species.

### Phyllosphere bacterial communities from the same plant species are relatively stable in different built environments

All factors (plant species and environment) and their interactions influenced the bacterial community structures (P < 0.05; Table 1). Non-metric multidimensional scaling (NMDS) ordination showed that bacterial communities formed discrete clusters according to plant species as well as the environment (Fig. S3). Plant species was the dominant factor that influenced the bacterial variation (13.7%), whereas the environment and its interaction with plant species explained 12.5% and 9.3% of the variation, respectively (Table 1). Pairwise comparison showed that each environment harbored a unique bacterial community structure (P_adjusted_ < 0.05). Similar to the bacterial communities, fungal communities also formed discrete clusters according to plant species as well as the environment (P < 0.05; Table 1, Fig. S3A-B). However, here the environment was the main factor that affected the fungal community structure (R^2^ = 25.6%) with a lower effect of the plant species (R^2^ = 8.6% and R^2^ = 12.5% respectively; Table 1). Pairwise comparisons showed that each sampling site harbored a unique fungal community structure (P_adjusted_ < 0.05).

**Table 1:** Statistical analysis of differences in beta diversity measures between plant species and environment.

**Factor**	**Microbial community similarities**	
	R^2^ value	P value
**Bacteria**		
Plant species (P)	0.137	0.001*
Environment (E)	0.125	0.001*
P × E	0.093	0.001*

**Fungi**		
Plant species (P)	0.086	0.001*
Environment (E)	0.256	0.001*
P × E	0.125	0.001*

**Archaea**		
Plant species (P)	0.023	0.589
Environment (E)	0.062	0.224
P × E	0.085	0.021*

*Significant differences (*p* ≤ 0.05) were assessed with the Adonis test.

Hierarchical clustering of the bacterial communities using the the Bray-Curtis dissimilarity matrix revealed two major clusters consisting of samples from the same plant species, regardless of the sampling location (Fig. S4A). Unlike the bacterial communities, the fungal communities showed a higher variation between samples from the same plant species obtained from different sampling locations (Fig. S4B). The results indicated that phyllosphere bacterial communities from the same plant species are relatively more stable in comparison to the fungal communities which were highly affected by the sampling locations.

In contrast to the bacterial and fungal fractions of the microbiome, there was no clear clustering of archaeal communities based on the factors tested in this study (Fig. S3C). Although the plant species and environment significantly influenced the archaeal community structure (P = 0.021), this factor only accounted for 8.5% of the variation (Table 1). An additional analysis was performed by separating all environments into individual sampling sites and subsequently assessing the effect of plant species. Plant species and environment showed an impact on the archaeal community structure (P = 0.019 and P = 0.005, respectively) and explained a total of 36.4% of the variation. For instance, the archaeal community structures in the private houses G and K, when compared to samples from the botanical garden, were significantly different (P < 0.05, Table S4). However, there was no significant difference in archaeal community structures between private house F when compared to samples from the botanical garden (P = 0.256). These results indicated a distinct variability between samples from private houses.

### Metagenomic analyses indicated frequent occurrence of bacterial genes involved in secondary metabolite biosynthesis

We further performed shotgun metagenome sequencing with representative samples from the botanical garden to explore functional aspects. The plants were grown in the same indoor environment for a long time under stable temperature and humidity, which reduces potential temporal influences of other factors on the microbiota. From the assembled *C. comosum* and *M. acuminata* metagenome contigs a total of 35,495 (min = 23,130, max = 53,952) and 23,442 (min = 14,295, max = 40,459) coding DNA sequences, respectively, were assigned to functional categories using the eggNOG database. We performed gene-centric analyses related to the production of secondary metabolites based on metagenome-assembled contigs. A total of 74 and 37 coding DNA sequences in the *C. comosum* metagenome were annotated as peptide synthetase and cytochrome P450 genes, respectively. These genes were also found in the *M. acuminata* metagenome; however, in a lower number (7 and 6 coding DNA sequences, respectively). Within the metagenomic data, specific genes which are involved in the biosynthesis of siderophores, namely Aerobactin (*iucC* and *iucD*) and Petrobactin (*asbA*) were detected in *Bacillus*-derived contigs. *PhzF-*like genes involved in the biosynthesis of the antibiotic phenazine were detected in *Sphingomonas*- as well as *Bacillus*-derived contigs.

**Fig. 2:**
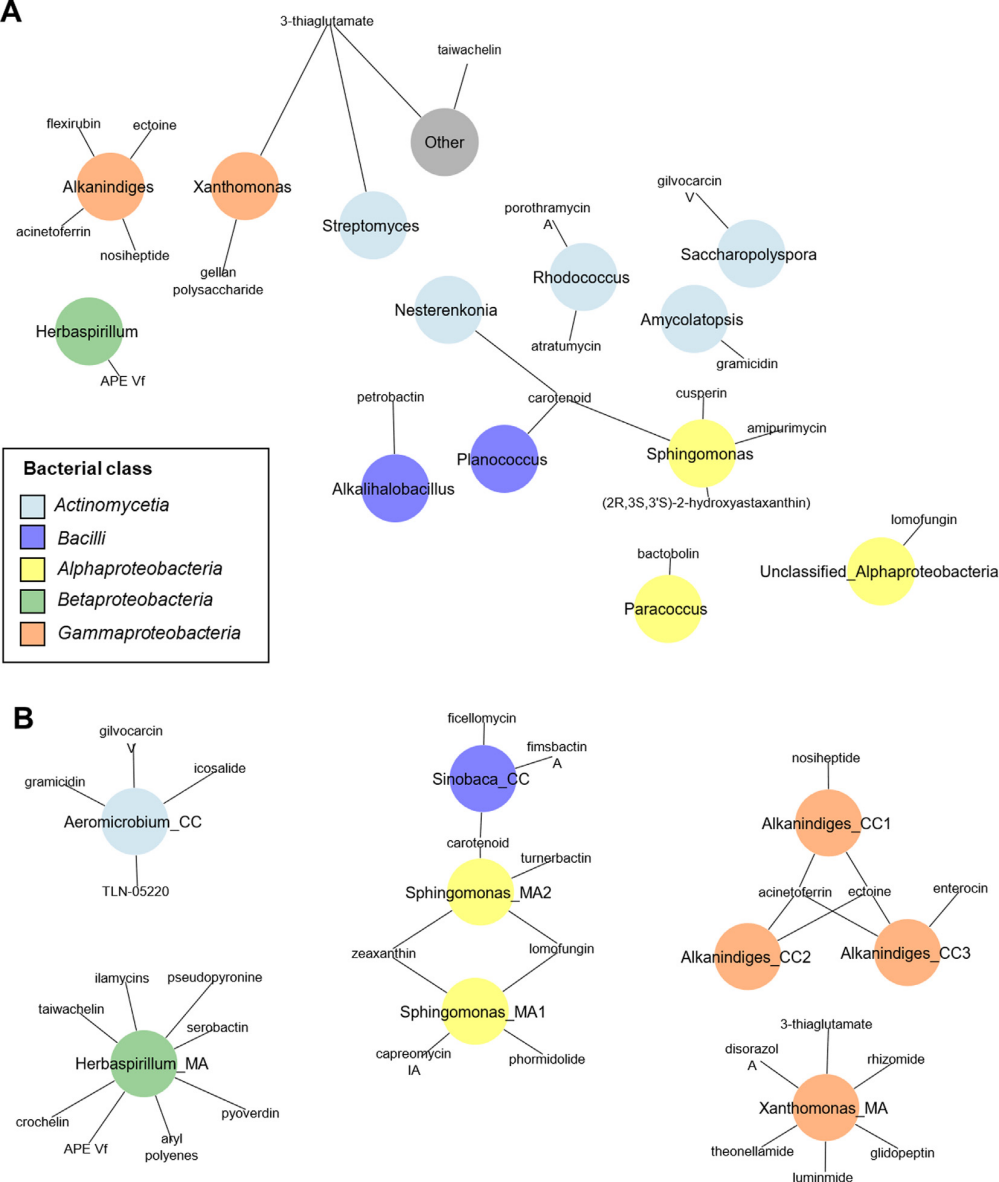
**Secondary metabolite gene clusters in metagenome-assembled contigs (A) and metagenome-assembled genomes (MAGs; B).** Secondary metabolite gene clusters were predicted using antiSMASH. Taxonomic information of contigs which contain the predicted secondary metabolite gene clusters was obtained using the Kaiju taxonomic classifier.

In a deepening analysis, we identified a total of 319 contigs that were inferred to secondary metabolite gene clusters according to processing with antiSMASH. A total of 34 contigs (*C. comosum:* 19 contigs and *M. acuminata:* 15 contigs) were identified as bacteria-derived contigs according to the Kaiju taxonomic classifier. Seven and five contigs were shown to encode acinetoferrin and carotenoid, respectively. We also identified several contigs that encode antimicrobial compounds such as amipurimycin, gramicidin, atratumycin, glivocarcins, and porothramycin (Fig. 2A). These contigs were assigned to to *Sphingomonas* (Alphaproteobacteria), *Rhodococcus*, *Amycolatopsis*, and *Saccharopolyspora* (*Actinomycetia*). Furthermore, we could recover 14 MAGs (completeness > 50% and contamination < 10%, Table S8). Within the genome collection, biosynthetic gene clusters were found in 9 MAGs (Fig. 2B). A number of antimicrobial compounds such as lomofungin, turnerbactin, and capreomycin was present in the *Sphingomonas* genome. Therefore, it can be assumed that bacteria within the indoor plant phyllosphere can produce a broad range of bioactive compounds potentially suppressing pathogens.

### Bacterial isolates from indoor plants were highly active against opportunistic human pathogens

**Fig. 3:**
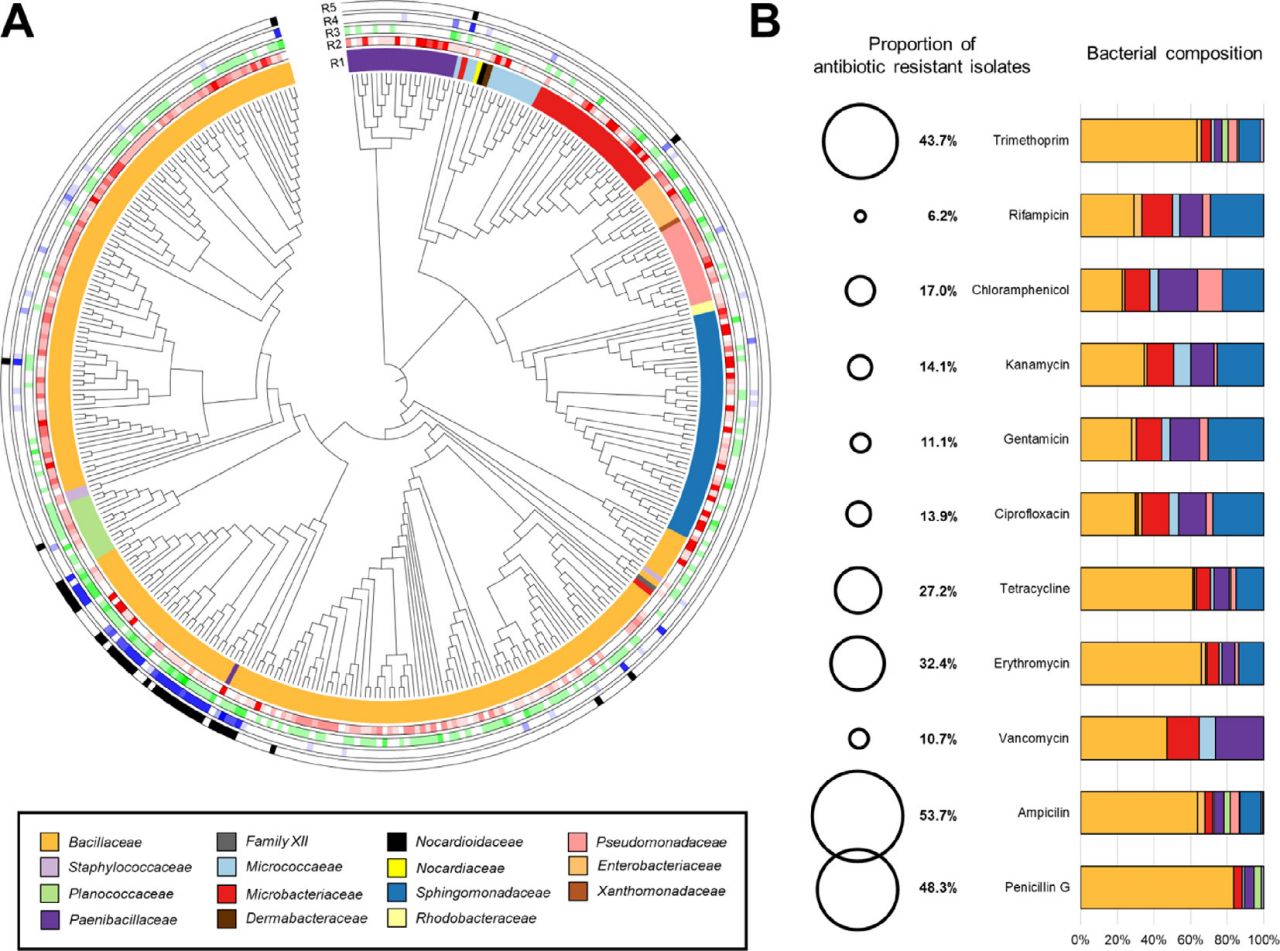
**Detailed taxonomic and functional assessment of the bacterial communities in the phyllosphere of the two model plants. A phylogenetic tree was constructed with partial 16S rRNA gene sequences of bacterial isolates from *Musa acuminata* and *Chlorophytum comosum* (A). Antibiotic resistance profiling of bacterial isolates and relative proportion of bacterial isolates that showed resistances to each of the tested antibiotics after normalization according to their their Gram classification (B).** The phylogenetic tree was visualized using interactive tree of life software (iTOL) based on sequence alignments using MUSCLE. The distance matrix was calculated with the maximum-likelihood algorithm. Ring 1 (R1) indicates bacterial taxonomy. Ring 2 (R2) indicates the number of resistances to antibiotics (dark red: higher number of resistances and light red: lower number of resistances). Ring 3 (R3) indicates biofilm synergism (dark green: higher number of synergistic interactions and light green: lower number of synergistic interactions). Ring 4 (R4) indicates antagonism against the implemented opportunistic pathogens (dark blue: higher number of antagonistic interactions and bright blue: lower number of antagonistic interactions). Ring 5 (R5) indicates bacterial isolates that were shown to produce biosurfactants (black).

In order to explore if phyllosphere bacteria can counteract opportunistic human pathogens, the isolates were implemented in targeted functional assays. We cultured bacterial isolates from the same samples that were used for shotgun-sequenced metagenomes. This approach allowed us to correlate screenings based on the metagenomic data and plate assays. We isolated and sequenced a total of 389 isolates (n = 264 from *C. comosum* and n = 125 from M. acuminata). Overall, the majority of the isolates was assigned to the bacterial family Bacillaceae (60.1%; n = 234), this was followed by Sphingomonadaceae (11.3%; n = 44), Microbacteriaceae (7.5%; n = 29), Paenibacillaceae (5.7%; n = 22%), and Pseudomonadaceae (4.1%; n = 16) (Fig. 3A).

A total of 45 isolates (out of 389 total isolates) demonstrated antagonistic activity against multiple opportunistic pathogens (Fig. 3A). The majority of these isolates (n = 38) was assigned to the genus *Bacillus* (Bacillaceae), whereas the rest of them was identified as *Microbacterium* (Microbacteriaceae, n = 2), *Sphingomonas* (Sphingomonadaceae, n = 1), *Paenibacillus* (Paenibacillaceae, n = 1), *Pseudarthrobacter* (Micrococcaceae, n = 1), and *Lysinibacillus* (Bacillaceae, n = 1). Overall, more antagonistic isolates against the tested opportunistic human pathogens were detected within the *C. comosum* culture collection (Fig. S5). Out of 264 isolates, 50 (18.9%) inhibited at least one model pathogen, whereas half of the isolate collection (n = 25) showed inhibition against five different pathogens. Almost the same fraction of isolates was found to inhibit *P. aeruginosa* (n = 40; 15.2 %), *E. faecium* (n = 39, 14.7 %), *S. maltophilia* (n = 34; 12.9 %) *S*. *haemolyticus* (n = 33; 12.5 %) and *E. coli* (n = 26; 9.8 %). Remarkably, there was no isolate in the culture collection with inhibitory effects against *A. baumanii*. We hypothesized that *A. baumanii* and the cultured bacterial isolates might be engaged in either neutral or even positive interactions. To test this hypothesis, we performed a correlation network analysis based on 16S rRNA gene fragment amplicons and found a positive correlation (P < 0.05, r = 0.519; Table S5) between cultured bacterial isolates including *Bacillus*, which was the most prevalent isolate (Fig. 3A) and *Acinetobacter* in the *C. comosum* dataset. Therefore, we assume that these taxa do not antagonise each other. A total of 125 bacteria were isolated from *M. acuminata* and 13 of them (10.4 %) showed an inhibitory effect against at least one pathogen. Most of them showed inhibition against *S. haemolyticus* (n = 10; 8 %) followed by *P. aeruginosa* (n = 6; 4.8 %), *E. faecium* (n = 4; 3.2 %), *E. coli* and *S. maltophilia* (n = 3; 2.4 %) and *A. baumanii* (n = 2; 1.5%).

Survival of opportunistic pathogens in built environments is often associated with biofilm formation*.* Hence, we were interested if there is a potential synergism/non-synergism with the phyllosphere-associated bacteria. A total of 196 (out of 389) isolates showed non-synergistic interactions in the co-cultured biofilm with the opportunistic pathogens (Fig. 3A). A significant reduction in biofilm formation was observed in co-cultures of indoor plant isolates with *E. faecium*, where non-synergistic effects were found for 91.2% (n = 114) of *M. acuminata* isolates and 97.2% (n = 256) for *C. comosum* (Fig. S6). A similar trend was observed for *S. maltophilia*, where the proportions of non-synergistic interactions for both plants were also relatively high (88 %, n = 110 for *M. acuminata* and 87.9%, n = 232 for *C. comosum*). In contrast to the non-synergistic effects with the other model pathogens, the results of the conducted assay indicated mostly synergistic (65.6%; n = 82) interactions between bacteria isolated from *M. acuminata* and *P. aeruginosa* in mixed species biofilms*.* This was mainly observed with isolates from *M. acuminata*, while only 28.4% (n = 77) of the bacteria originating from *C. comosum* showed synergistic interactions with *P. aeruginosa*. Interestingly, correlation network analysis based on the 16S rRNA gene fragment amplicons revealed a positive correlation (Table S5 P < 0.05, r = 0.605) between *Bacillus* (as representative of prevalent isolates; Fig. 3A) and *Pseudomonas* in the *M. acuminata* dataset. The overall findings suggest that mainly non-synergistic interactions prevail in mixed species biofilms of phyllosphere-associated bacteria and opportunistic human pathogens.

The high prevalence of antagonism can be partially explained by the ability of bacterial isolates to produce biosurfactants. Most of the isolates that inhibited multiple opportunistic pathogens were later shown to produce these compounds (Fig. 3A). For the *M. acuminata* isolates, five out of 125 isolates showed positive results in the drop collapse assay. Interestingly four of them also showed inhibitory activity against the implemented pathogens. A similar tendency was observed for *C. comosum*, where 33 of 264 isolates were able to produce biosurfactants and 31 of them also showed an inhibitory effect against the pathogens.

### Comparison with abiotic surfaces in the built environment revealed that indoor plants harbor a distinct resistome composition

**Fig. 4:**
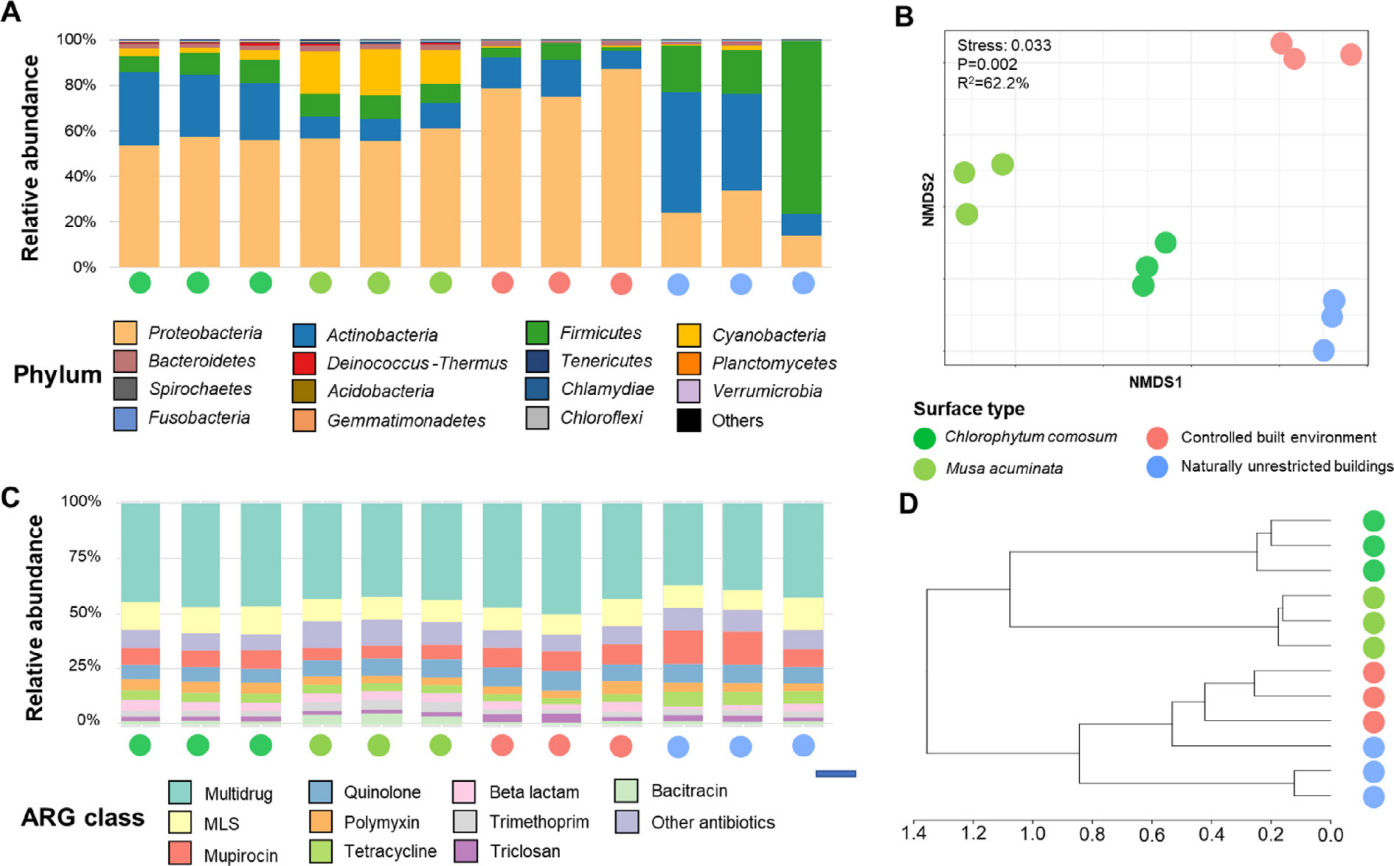
**Comparative assessment of community structures and occurrence of antibiotic resistance genes in plant and abiotic surface metagenomes.Composition and clustering were assessed for the different bacterial communities (A and B) as well as for the antibiotic resistance genes (C and D) detected in plant and abiotic surface metagenomes**. The bacterial taxonomic compositions and abundances within metagenomes were obtained with Kraken2 and Bracken, respectively. Bacterial community clustering was visualized using a non-metric multidimensional scaling (NMDS) plot based on a Bray-Curtis dissimilarity matrix (B). Hierarchical clustering of antibiotic resistance gene composition within the metagenomic datasets is based on a Bray-Curtis dissimilarity matrix (D).

We further aimed to investigate how specific the phyllosphere microbiome of the two model plants is with respect to indoor abiotic surfaces. The indoor plant phyllosphere bacterial community structures of *C. comosum* and *M. acuminata* were compared to those of abiotic surfaces from naturally unrestricted buildings and controlled built environments. Bacterial communities that were present in all samples showed a high proportion of Proteobacteria, Firmicutes*,* and Actinobacteria that contributed to more than 75% of the total bacterial communities (Fig. 4A) indicating a congruent result with the amplicon sequencing data. Comparisons of different groups indicated that Proteobacteria were relatively higher abundant in the controlled built environment (74–78%) in comparison to the naturally unrestricted buildings and the plant surfaces (14–60%), whereas Firmicutes showed the opposite trend. Beta diversity analysis indicated a significantly different clustering of bacterial community structures in each of the analysed environments (P = 0.002, R^2^ = 62.2%; Fig. 4B). Samples from naturally unrestricted buildings clustered closer to plant surfaces. Linear discriminant analysis effect size (LEfSe) analysis confirmed that Firmicutes were more prevalent in plant samples and naturally unrestricted buildings whereas the highest prevalence of Proteobacteria was detected in the controlled built environment (Fig. S7). Overall, it was confirmed that indoor plants harbor a distinct bacterial community in comparison to the abiotic built environment.

Within the phyllosphere metagenomes of *C. comosum* and *M. acuminata* a relatively low proportion of reads (0.052 – 0.190%) was assigned to 240 and 244 antimicrobial resistance genes (ARGs), respectively. These ARGs were subsequently assigned to 25 and 24 antibiotic classes. Multidrug resistance was the most prevalent resistance category; it was followed by resistances against macrolide, lincosamide and streptogramin (MLS), and mupirocin antibiotics (Fig. 4C). The ARG richness in the *C. comosum* and *M. acuminata* phyllospheres were lower (n = 245 and 239, respectively) but not statistically different in comparison to the ARG richness in the controlled built environment (n = 256) and naturally unrestricted buildings (n = 243, P = 0.133). Beta diversity analysis indicated that the sample type (plant surfaces and non-biological surfaces) explained 68.8% of the ARG composition (P = 0.001). Hierarchical clustering resulted in two major clusters that were divided into plant samples and abiotic surfaces (Fig. 4D) indicating distinct differences in their ARG compositions. A complementary analysis using the Mantel test indicated a significant correlation between bacterial communities and ARG composition (r = 0.612, P = 0.001).

In a deepening analysis based on LEfSe, multidrug resistance genes were found to be more prevalent in the controlled built environment (Fig. S8A), this was followed by the plant surfaces. Three antibiotic classes, i.e. mupirocin, aminicoumarin, and quinolone were more prevalent in the built environment in comparison to the plant surfaces, whereas trimethoprim and chloramphenicol followed the opposite trend. Analyses conducted with the Kaiju taxonomic classifier, indicated that the majority of ARGs carriers on the plant surfaces belonged to Gammaproteobacteria (34.7–43.7%) and Bacilli (24.3–28.1%), whereas in the controlled built environment most of the carriers were assigned to Gammaproteobacteria (52.4%, Fig. S8B). The ARG carriers in naturally unrestricted buildings were dominated by Gammaproteobacteria and Actinobacteria with a similar relative abundance (32.1% and 33.8%, respectively). It was concluded that the bacterial community composition was the main determinant for ARG occurrence in the different sample types.

### Cultivation-dependent studies confirmed the presence of multi-resistant bacteria

Within the whole collection, 75% of the isolates were resistant against at least one antibiotic (Fig. 3A). Moreover, 115 isolates showed resistance against at least three antibiotic classes and 45 isolates showed resistance against all tested antibiotic classes. The most common resistances were against ampicillin (53.7%), penicillin G (48.3%), trimethoprim (43.7%), erythromycin (32.4%), and tetracycline (27.2%) (Fig. 3B). The relative proportion of antibiotic resistant bacteria was calculated after normalizing the antibiotic resistance profiles according to the Gram classification of the respective bacteria (Fig. 3B). Antibiotic resistant isolates were mostly assigned to *Bacillus* due to the high prevalence of this taxon in the culture collection. In addition, multiple isolates (n = 13) that showed resistance against all tested antibiotics were identified as *Sphingomonas* (Sphingomonadaceae). A high proportion of these showed a distinct resistance against three antibiotics, namely gentamicin, ciprofloxacin, and rifampicin. Moreover, a high number of *Paenibacillus* isolates (n = 8) showed resistance against vancomycin. In total, 29 isolates from *M. acuminata* and 67 from *C. comosum* did not show any resistance against the employed antibiotics. Interestingly, isolates that showed antagonism towards the opportunistic pathogens and an ability to produce biosurfactants harbored overall fewer resistances to antibiotics (Fig. 3A).

## Discussion

In the present study, in which two indoor plant species from different built environments were analyzed using a multiphasic approach, we made three key observations: (i) the plant species as well as the built environment influenced phyllosphere microbiomes of indoor plants; bacterial communities were mainly shaped by the plant species, while fungal and archaeal communities were primarily shaped by the built environment; (ii) irrespective of the environment, plants harbored a plant-specific structural and functional core microbiome, and (iii) phyllosphere bacteria can potentially act as a protective shield against opportunistic human pathogens. Based on our findings we propose a model that encompasses “stability and establishment”, “antagonism” and “persistence” as distinctive components of an intrinsic defense line of the indoor plant phyllosphere microbiota against opportunistic human pathogens. The intrinsic resistome, a large repertoire of bioactive compounds, and auxiliary functions hinder their establishment in the phyllosphere and thus serve as a natural infection barrier. Once transferred indoors, opportunistic pathogens must establish and multiply; those that land on leaf surfaces are counteracted by the phyllosphere microbiota. In previous studies it was shown that plant-associated bacteria were integrated in the indoor room microbiome [5], [7]. This opens interesting possibilities to apply probiotics for a healthy and stable indoor microbiome. In addition to these new aspects, our data are in line with previous observations related to the plant and specifically the phyllosphere microbiome, e.g., the general composition of bacterial, fungal, and archaeal communities and a predominance of Alpha- and Gammaproteobacteria, Firmicutes, Dothideomycetes*,* and Thaumarchaeota.

This study provides the first detailed insights on how commonly occurring bacterial members of the indoor plant phyllosphere may rely on their repertoire of genes related to bioactive compound production to counteract opportunistic human pathogens. Bioactive properties of plant-associated bacteria, *e.g*. antibiotic production and antagonistic activity against human opportunistic pathogens, were previously reported for medicinal plants [68], [69], [70]. To the best of our knowledge, there is no study that reports antagonistic activity of bacteria isolated from indoor plants against opportunistic human pathogens. Genes encoding multi-enzyme complexes of non-ribosomal peptide synthetases and cytochrome P450 which catalyse the production of numerous natural products with antimicrobial, phytotoxic and antiviral activities [71], [72] were abundant within the analyzed data in the present study*.* Specific genes that encode antimicrobial compounds, *i.e*. phenazine and lomofungin, were recovered from contigs derived from *Bacillus* and *Sphingomonas*. Phenazine is a well-known antibacterial agent [73], [74], [75]. Hence, we argue that the strong antagonistic activity against opportunistic human pathogens that was observed with bacterial isolates is due to the production of bioactive compounds. Moreover, we observed a high occurrence of biosurfactant-producing bacteria, which are known to effectively disrupt biofilm formation [76], [77]. Bacteria that normally colonize the same habitat show more frequently synergistic effects in mixed-community biofilm formation, which is indicative for an adaptive response to long-term co-existence [78]. This might also be the reason why mostly negative effects were observed in biofilms consisting of human pathogens and phyllosphere colonizers in this study. Taken altogether, the present study indicated that bacterial members of the phyllosphere of indoor plants can potentially counteract opportunistic human pathogens by relying on various well-known modes of action.

Detailed analyses revealed a differential influence of plant species and built environment on bacterial, archaeal and fungal community structures; bacterial communities were substantially more plant-specific than the other microbiota members. This underlines the intimate plant-bacteria relation found in this study and earlier for the rhizosphere and endosphere microbiota [79], [80]. Fungal and archaeal communities in built environments are also known to be substantially affected by outdoor air and indoor occupants [81], [82]. Moreover, fungi are easily dispersed through airborne spores [83] which likely influence the variability of fungal communities in the phyllosphere of indoor plants observed between different built environments. On the other hand, the plant species was previously suggested as a major driver of bacterial community structure and richness in the indoor plant phyllosphere, which is likely due to differences in leaf morphology and host metabolism [6], [84], [85]. The comparatively lower variation of bacterial community structures between different built environments suggests that plants can form a more stable bacterial core community; in the present case consisting of *Bacillus*, *Sphingomonas*, and *Microbacterium.* These core members demonstrated a common functional role in terms of their potential to counteract opportunistic human pathogens. We suggest that this stable community is a key requirement to counteract invasion and expansion of opportunistic pathogens in the phyllosphere of indoor plants. The unexpected synergistic effect between *M. acuminata* associated bacteria and *Pseudomonas* might be explainable by the high prevalence of members of the genus *Pseudomonas* in plant-associated habitats [86], [87]. This also applies to the present study, where *Pseudomonas* was identified as a component of the *M. acuminata* core microbiome. We hypothesize that certain bacteria might be recognized as plant inhabitants even if they originate from clinical settings and therefore engage in partially synergistic interactions that were observed in the present study.

When the microbiome of the indoor plant phyllosphere was compared with that of abiotic indoor surfaces, the former demonstrated a broad range of antimicrobial resistance features within the bacterial community. Differences in ARG diversity and composition between the indoor plant metagenomes and the abiotic surfaces likely reflect the distinct microbial communities in each environment. Most of the ARG-harboring bacteria in the indoor plant phyllosphere are not considered as human pathogens [88]. Furthermore, because most of the opportunistic human pathogens used as models in the present study were antagonized by the natural phyllosphere bacterial community, ARG transmission from the natural phyllosphere bacteria is less likely. We hypothesize that intrinsic resistomes are required by indoor plant-associated bacteria to survive in a considerably hostile environment such as the phyllosphere. Certain differences were observed when the phyllosphere resistomes were compared with resistomes of the implemented abiotic surfaces in built environments. It can be expected that the high prevalence of multidrug resistance genes in the controlled built environment is likely shaped by different cleaning regimes as already concluded in the original study from which the datasets were obtained [9]. Interestingly, phyllosphere bacteria were shown to carry a high abundance of multidrug resistance genes in comparison to the naturally unrestricted buildings. *Musa acuminata* as well as the genus *Chlorophytum* are known to produce distinct antimicrobial compounds [89], [90] that may have detrimental effects on bacteria and consequently shape the microbial community structure. Hence, the presence of antibiotic resistance genes might be required for detoxification of plant metabolites [91], [92]. We suggest that this intrinsic resistomes are part of co-evolution between plants and their associated bacteria and are needed for them to persist in the phyllosphere.

Here we showed that naturally occurring phyllosphere bacteria can potentially act as a protective shield against opportunistic human pathogens. However, the experimental design did not allow us to experimentally test the effect of the microbiota of indoor plants on the diversity and abundance of opportunistic pathogens in the built environment. This will require large-scale experiments and longitudinal observations to investigate whether the introduction of indoor plants can sufficiently increase microbial diversity and limit the spread of opportunistic pathogens in the built environment. If confirmed, this would be interesting for developing plant microbiome-derived indoor probiotics, which can be introduced directly or indirectly to establish a healthy microbiome in built environments. Such measueres are especially important to avoid healthcare-associated infections, which are a significant cause of illness and death in hospitals and intensive care units.

## Conclusions

This study provides key insights into the function of naturally occurring microbes on the leaf surface of the common indoor plants *C. comosum* and *M. acuminata* and their potential implications for human health. It was shown that phyllosphere microbiomes are mainly influenced by the plant species and the built environment in which the plant is grown. In addition, the bacterial community showed higher congruence within each of the plant species compared to the archaeal and fungal communities of the same plants. We demonstrated that phyllosphere bacteria with their distinct properties can potentially counteract opportunistic human pathogens, which should be further explored in the future.

## Declaration of Competing Interest

The authors declare that they have no known competing financial interests or personal relationships that could have appeared to influence the work reported in this paper.
