# Supplementary material for: Phyllosphere-associated microbiota in built environment: Do they have the potential to antagonize human pathogens?
Source: J Adv Res. 2022 Feb 12;43:109–21. doi: 10.1016/j.jare.2022.02.003 (PMC9811327; doi:10.1016/j.jare.2022.02.003)
Supplement: Supplementary data 1 [file mmc1.docx]

**Phyllosphere-associated microbiota in built environment:**

**Do they have the potential to antagonize human pathogens?**

**Wisnu Adi Wicaksono^a^, Tamara Reisenhofer-Graber ^a^, Sabine Erschen^a^**, **Peter Kusstatscher^a^, Christian Berg^b^, Robert Krause^cd^,** **Tomislav Cernava^ad^, Gabriele Berg^adef^**

*^a^Institute of Environmental Biotechnology, Graz University of Technology, Graz, Austria*

*^b^Institute of Plant Sciences, Karl-Franzens-University, Graz, Austria*

*^c^Department of Internal Medicine, Medical University of Graz, Graz, Austria*

*^d^BioTechMed Graz, Inter-university Cooperation Platform, Graz, Austria*

^e^Leibniz Institute for Agricultural Engineering and Bioeconomy Potsdam, Potsdam, Germany

^f^Institute for Biochemistry and Biology, University of Potsdam, Potsdam, Germany

**Authors’ email addresses:** reisenhofer-graber@student.tugraz.at, sabine.erschen@tugraz.at, peter.kusstatscher@tugraz.at, christian.berg@uni-graz.at, robert.krause@medunigraz.at, gabriele.berg@tugraz.at

**Short title:** Indoor plant microbiomes

**Submitted to:** Journal of Advanced Research


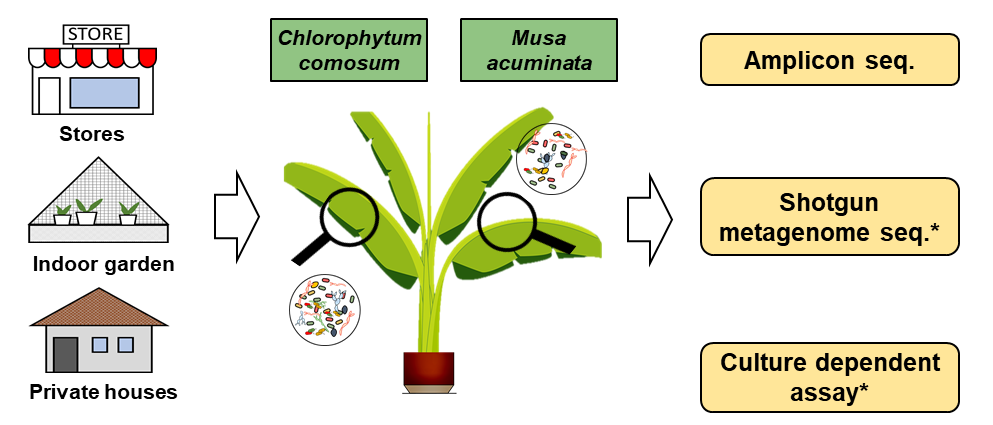


**Figure S1. Sampling strategy and experimental workflow that were implemented in this study. Indoor plant samples were collected from three built environments including stores, glass houses of an indoor garden (Graz Botanical Garden), and private houses (details in Table S1)**. All of the samples were subjected for amplicon sequencing of bacterial, fungal, and archaeal marker genes. Shotgun metagenome sequencing and culture dependent assays (labelled with an asterisk) were performed only for samples from the botanical garden.


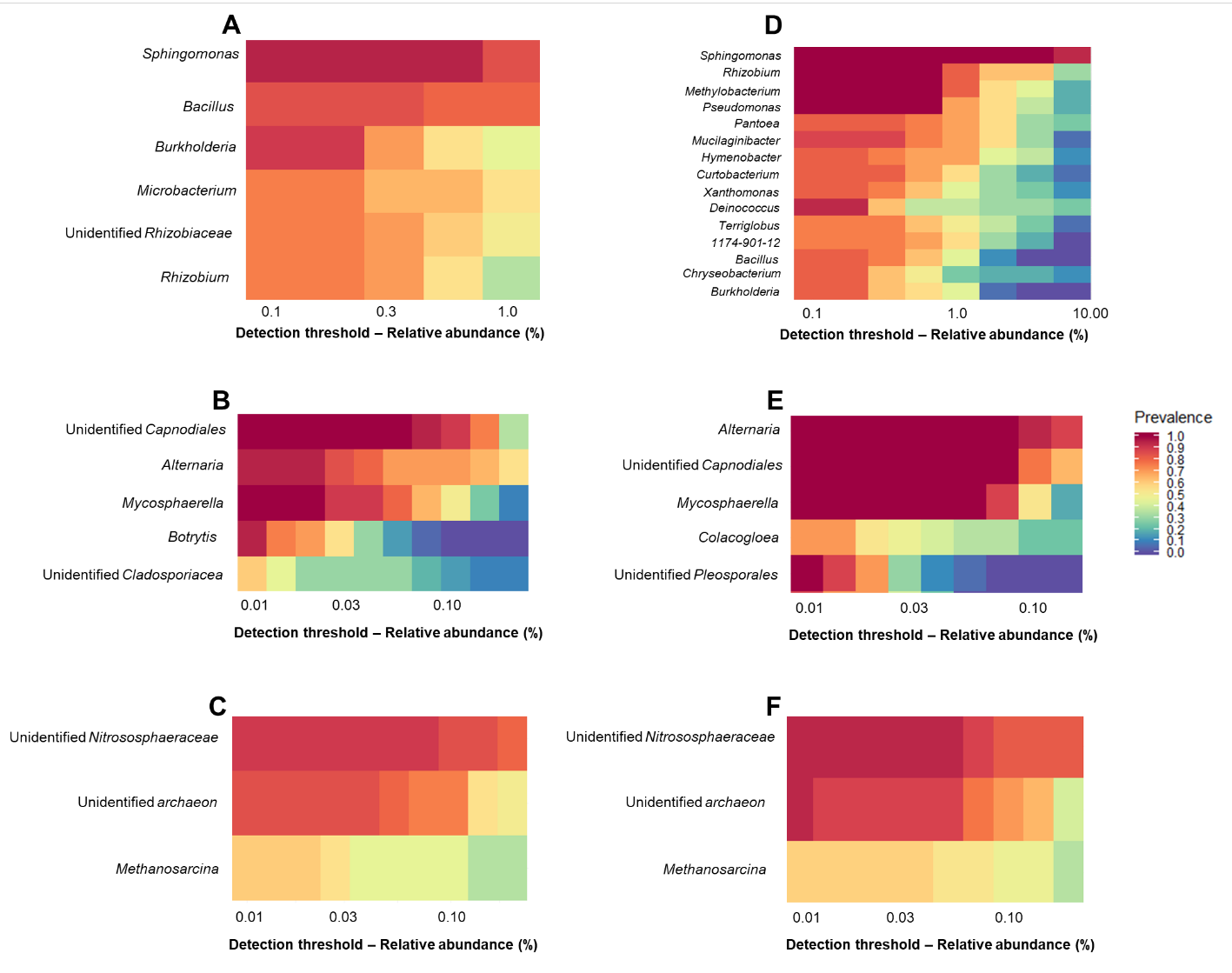


**Figure S2. Core microbiome of the *Chlorophytum* *comosum* (A-C) and *Musa acuminata* (D-F) phyllosphere determined by amplicon sequencing of marker genes.** The prevalent amplicon sequencing variants were assigned at the bacterial (A and D), fungal (B and E), and archaeal (C-F) genus level and their prevalence in the core microbiomes was visualized in heatmap plots.


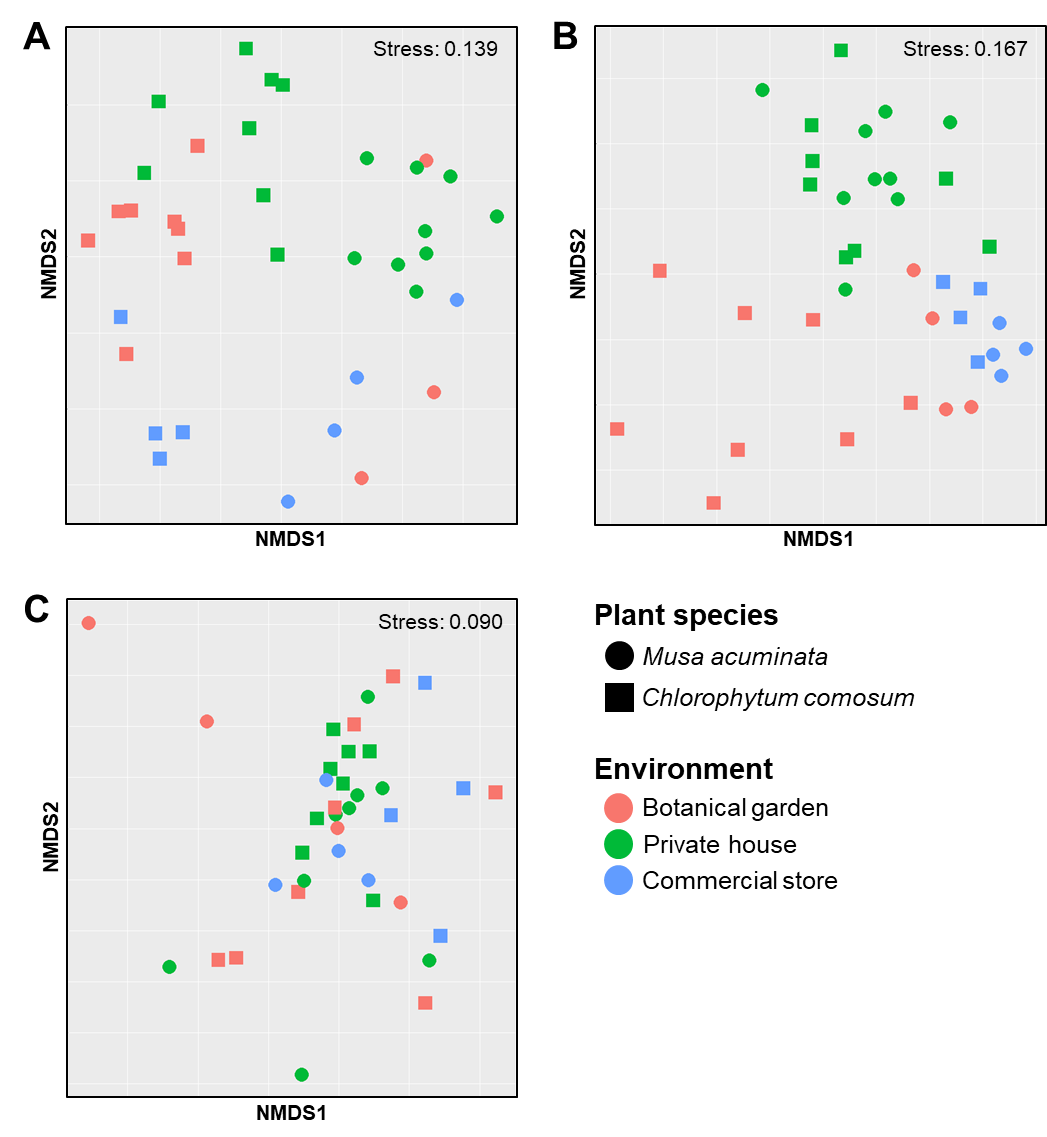


Figure S3. Non-metric multidimensional scaling (NMDS) ordination of bacterial (A), fungal (B) and archaeal (C) communities. The analysis is based on a Bray-Curtis dissimilarity matrix that was obtained for each dataset. The sample type and sampling location are indicated by different shapes and colors.


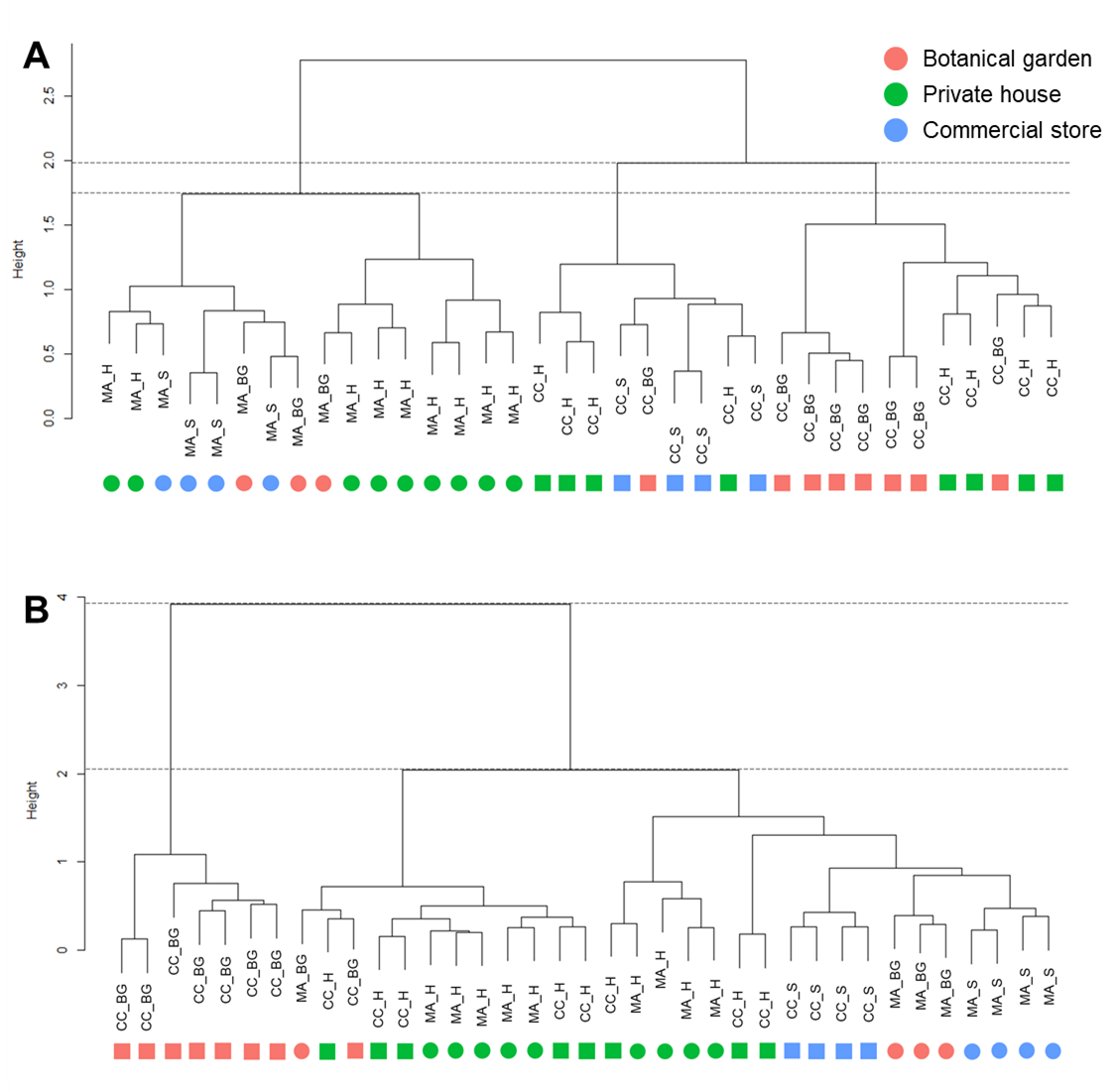


**Figure S4. Hierarchical clustering of bacterial (A) and fungal (B) community structures of the indoor plant phyllosphere based on a Bray-Curtis dissimilarity matrix.** Sample codes indicate the plant species, namely CC: *Chlorophytum* *comosum,* MA: *Musa acuminata* and sampling location, namely BG: botanical garden, H: private house, S: commercial store.


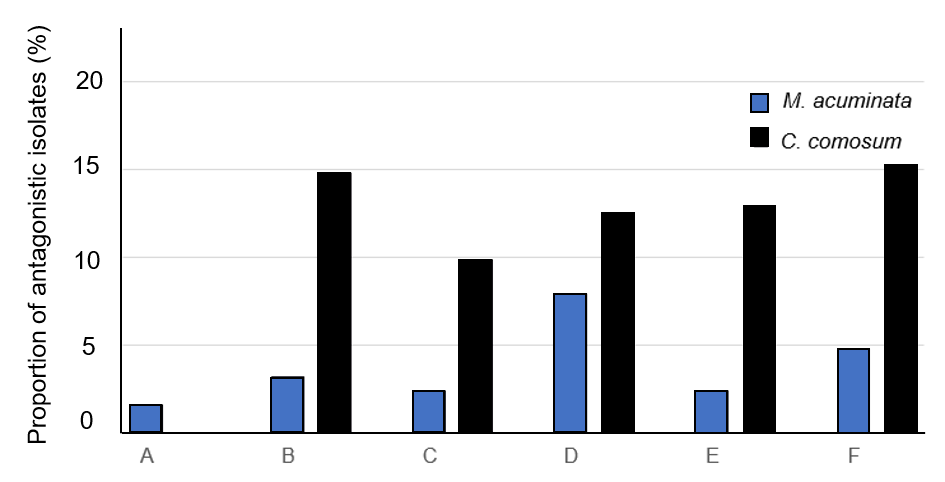


**Figure S5. Proportion of bacteria that showed antagonist effects against human opportunistic pathogens.** (A) *Acinetobacter baumanii*, (B) *Enterococcus faecium*, (C) *Escherichia coli*, (D) *Staphylococcus haemolyticus*, (E) *Stenotrophomonas maltophilia* and (F) *Pseudomonas aeruginosa*.


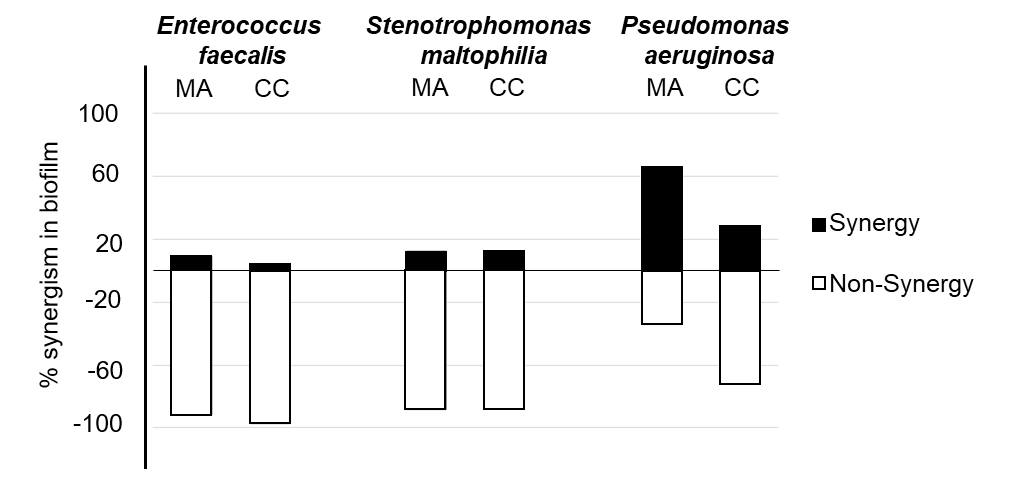


**Figure S6. Percentage of synergism in mixed biofilm screening of bacterial isolates from the indoor plants *Chlorophytum comosum* and *Musa acuminata*.** Synergistic interactions in mixed biofilm were performed by co-culturing human opportunistic pathogens (*Enterococcus faecalis* or *Stenothrophomonas maltophilia* or *Pseudomonas aeruginosa*) and indoor plant associated bacteria. MM: *Musa acuminata*, CC: *Chlorophytum comosum*.


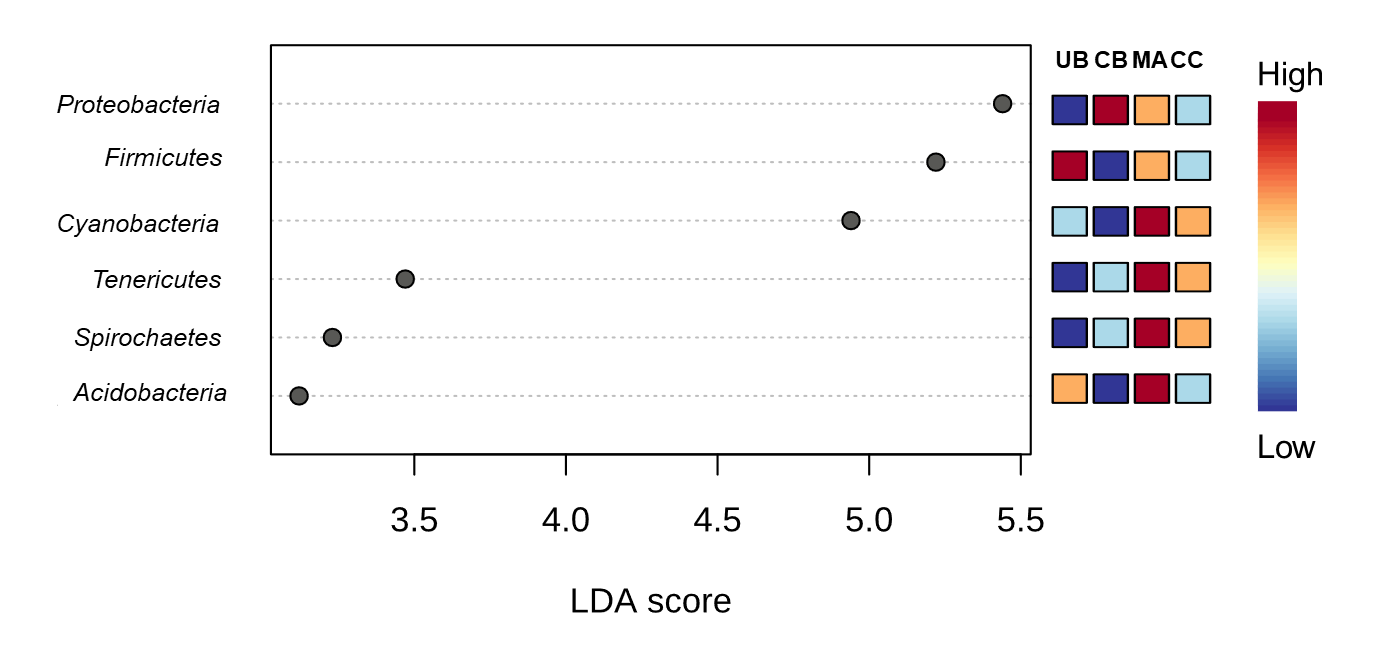


**Figure S7. LEfSe (Linear discriminant analysis effect size) was implemented to identify bacterial phyla that were differentially abundant in plant and abiotic surfaces.** UB: unrestricted buildings, CB: controlled built environment, MA: phyllosphere of *Musa acuminate*, CC: phyllosphere of *Chlorophytum* *comosum*.


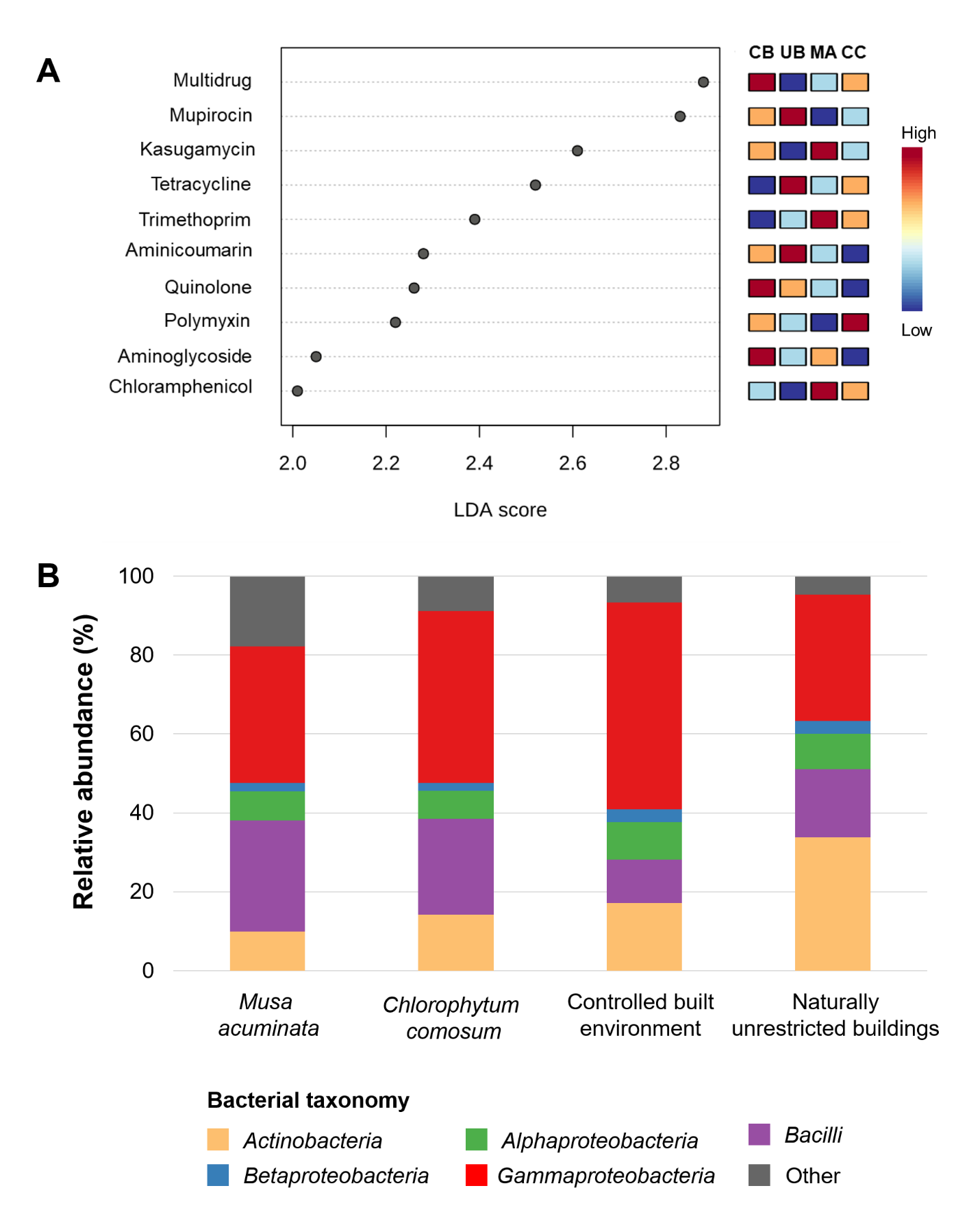


**Figure S8. LEfSe (Linear discriminant analysis effect size) was used to identify the antibiotic resistance classes that were differentially abundant (A). The relative abundance of antibiotic resistance carriers from the plant and abiotic surface metagenomic datasets was assessed (B).** UB: unrestricted buildings, CB: controlled built environment, MA: phyllosphere of *Musa acuminate*, CC: phyllosphere of *Chlorophytum* *comosum*.

Table S1. Sample overview for *Musa acuminata* (dwarf banana) and *Chlorophytum comosum* (spider plant) samples from three different built environments.

| Sample ID | Plant species | Built environment |
| --- | --- | --- |
| B1 | Dwarf banana | Botanical garden |
| B2 | Dwarf banana | Botanical garden |
| B3 | Dwarf banana | Botanical garden |
| B4 | Dwarf banana | Botanical garden |
| B5 | Dwarf banana | House G |
| B6 | Dwarf banana | House G |
| B7 | Dwarf banana | House G |
| B8 | Dwarf banana | House G |
| B9 | Dwarf banana | House G |
| B10 | Dwarf banana | House F |
| B11 | Dwarf banana | House F |
| B12 | Dwarf banana | House K |
| B13 | Dwarf banana | House K |
| B14 | Dwarf banana | Store 1 |
| B15 | Dwarf banana | Store 1 |
| B16 | Dwarf banana | Store 2 |
| B17 | Dwarf banana | Store 2 |
| L1 | Spider plant | Botanical garden |
| L2 | Spider plant | Botanical garden |
| L3 | Spider plant | Botanical garden |
| L4 | Spider plant | Botanical garden |
| L5 | Spider plant | Botanical garden |
| L6 | Spider plant | Botanical garden |
| L7 | Spider plant | Botanical garden |
| L8 | Spider plant | Botanical garden |
| L9 | Spider plant | House G |
| L10 | Spider plant | House G |
| L11 | Spider plant | House F |
| L12 | Spider plant | House F |
| L13 | Spider plant | House F |
| L14 | Spider plant | House F |
| L15 | Spider plant | House K |
| L16 | Spider plant | House K |
| L17 | Spider plant | Store 1 |
| L18 | Spider plant | Store 1 |
| L19 | Spider plant | Store 2 |
| L20 | Spider plant | Store 2 |

Table S2. Overview of the read statistics in the amplicon sequencing experiments for each sample and all three targeted sampling locations.

| Sample ID | Bacteria | | | Fungi | | | Archaea | | |
| --- | --- | --- | --- | --- | --- | --- | --- | --- | --- |
|  | Raw quality sequences | Filtered quality sequences | % non-target sequences | Raw quality sequences | Filtered quality sequences | % non-target sequences | Raw quality sequences | Filtered quality sequences | % non-target sequences |
| B1 | 168,805 | 3,960 | 97.7 | 10,590 | 7,564 | 28.6 | 24,477 | 24,381 | 0.4 |
| B2 | 5,915 | 893 | 84.9 | 25,155 | 15,000 | 40.4 | 6,485 | 6,485 | 0 |
| B3 | 9,358 | 427 | 95.4 | 31,455 | 20,598 | 34.5 | 1,841 | 1,841 | 0 |
| B4 | NA | NA | NA | 17,587 | 8,349 | 52.5 | 4,187 | 4,187 | 0 |
| B5 | 58,367 | 1,657 | 97.2 | 10,982 | 5,605 | 49 | 21,577 | 21,534 | 0.2 |
| B6 | 59,371 | 3,682 | 93.8 | 9,576 | 5,001 | 47.8 | 21,476 | 21,476 | 0 |
| B7 | 128,904 | 4,272 | 96.7 | 4,867 | 2,711 | 44.3 | 2,976 | 2,823 | 5.1 |
| B8 | 91,587 | 1,637 | 98.2 | 12,443 | 5,827 | 53.2 | 23,399 | 23,236 | 0.7 |
| B9 | 256,642 | 3,778 | 98.5 | 15,272 | 6,841 | 55.2 | 12,652 | 11,608 | 8.3 |
| B10 | 291,632 | 8,536 | 97.1 | 12,375 | 4,787 | 61.3 | 27,912 | 27,879 | 0.1 |
| B11 | 171,744 | 7,931 | 95.4 | 9,081 | 3,940 | 56.6 | 14,686 | 14,679 | 0 |
| B12 | 149,624 | 6,428 | 95.7 | 15,909 | 8,354 | 47.5 | 19,003 | 18,993 | 0.1 |
| B13 | 157,086 | 33,685 | 78.6 | 9,357 | 1,865 | 80.1 | 6,819 | 6,791 | 0.4 |
| B14 | 60,126 | 6,005 | 90 | 7,559 | 4,178 | 44.7 | 17,330 | 17,218 | 0.6 |
| B15 | 251,084 | 16,302 | 93.5 | 8,961 | 5,853 | 34.7 | 34,026 | 33,964 | 0.2 |
| B16 | 10,117 | 679 | 93.3 | 14,240 | 10,483 | 26.4 | 26,064 | 21,412 | 17.8 |
| B17 | 194,168 | 8,010 | 95.9 | 13,456 | 9,015 | 33 | 51,634 | 51,500 | 0.3 |
| L1 | 22,920 | 5,567 | 75.7 | 23,386 | 9,137 | 60.9 | 32,649 | 32,649 | 0 |
| L2 | 13,439 | 6,047 | 55 | 13,273 | 4,332 | 67.4 | 22,665 | 22,665 | 0 |
| L3 | 5,545 | 787 | 85.8 | 5,924 | 4,261 | 28.1 | 15,552 | 15,552 | 0 |
| L4 | 6,693 | 2,994 | 55.3 | 8,428 | 5,651 | 32.9 | 11,480 | 11,480 | 0 |
| L5 | 13,005 | 1,881 | 85.5 | 12,771 | 6,651 | 47.9 | 12,971 | 12,962 | 0.1 |
| L6 | 3,505 | 1,237 | 64.7 | 25,681 | 11,206 | 56.4 | 10,094 | 10,094 | 0 |
| L7 | 12,138 | 4,293 | 64.6 | 43,172 | 23,898 | 44.6 | 9,116 | 9,116 | 0 |
| L8 | 9,594 | 3,178 | 66.9 | 29,497 | 15,125 | 48.7 | 5,128 | 5,128 | 0 |
| L9 | 3,648 | 1,547 | 57.6 | 6,546 | 4,942 | 24.5 | 11,316 | 11,316 | 0 |
| L10 | 10,522 | 5,874 | 44.2 | 13,296 | 10,680 | 19.7 | 18,084 | 18,084 | 0 |
| L11 | 11,357 | 2,885 | 74.6 | 14,977 | 11,753 | 21.5 | 24,540 | 24,540 | 0 |
| L12 | 3,546 | 1,203 | 66.1 | 6,510 | 4,558 | 30 | 13,286 | 13,286 | 0 |
| L13 | 6,093 | 2,024 | 66.8 | 8,136 | 5,704 | 29.9 | 17,343 | 17,340 | 0 |
| L14 | 8,527 | 2,896 | 66 | 14,906 | 11,201 | 24.9 | 13,581 | 13,581 | 0 |
| L15 | 10,622 | 4,022 | 62.1 | 12,637 | 7,608 | 39.8 | 16,373 | 16,373 | 0 |
| L16 | 6,414 | 5,054 | 21.2 | 8,566 | 4,635 | 45.9 | 13,304 | 13,304 | 0 |
| L17 | 10,709 | 6,596 | 38.4 | 13,350 | 5,772 | 56.8 | 17,646 | 17,613 | 0.2 |
| L18 | 5,284 | 2,522 | 52.3 | 8,990 | 5,889 | 34.5 | 21,113 | 21,090 | 0.1 |
| L19 | 5,028 | 1,915 | 61.9 | 8,341 | 5,265 | 36.9 | 12,803 | 12,508 | 2.3 |
| L20 | 10,647 | 3,757 | 64.7 | 13,608 | 7,602 | 44.1 | 32,573 | 32,508 | 0.2 |

Table S3. Antibiotic resistance screening against 11 different antimicrobial substances.

| Antibiotic | Antibiotic class | Manufacturer | Spectrum |
| --- | --- | --- | --- |
| Benzylpenicillin (Penicillin G) | β-lactam | Roth, Germany | Gram + |
| Ampicillin | β-lactam | Roth, Germany | Gram +/ - |
| Vancomycin | Glycopeptide | Sigma-Aldrich, USA | Gram + |
| Erythromycin | Macrolide | Roth, Germany | Gram +/ - |
| Tetracycline | Tetracycline | Merck, German | Gram +/ - |
| Ciprofloxacin | Fluoroquinolone | Sigma-Aldrich, USA | Gram - |
| Gentamicin | Aminoglycoside | Roth, Germany | Gram +/ - |
| Kanamycin | Aminoglycoside | Roth, Germany | Gram +/ - |
| Chloramphenicol | Other | Roth, Germany | Gram +/ - |
| Rifampicin | Other | Duchefa Biochemie, Netherland | Gram +/ - |
| Trimethoprim | Other | Sigma-Aldrich, USA | Gram +/ - |

**Table S4. Permutational Multivariate Analysis of Variance: pairwise comparisons between individual sampling sites.**

| Pairwise comparison | P value |
| --- | --- |
| Botanical garden vs House G | 0.041 |
| Botanical garden vs House F | 0.256 |
| Botanical garden vs House K | 0.023 |
| Botanical garden vs Store 1 | 0.608 |
| Botanical garden vs Store 2 | 0.917 |
| House G vs House F | 0.227 |
| House G vs House K | 0.005 |
| House G vs Store 1 | 0.437 |
| House G vs Store 2 | 0.424 |
| House F vs House K | 0.049 |
| House F vs Store 1 | 0.924 |
| House F vs Store 2 | 0.387 |
| House K vs Store 1 | 0.150 |
| House K vs Store 2 | 0.109 |
| Store 1 vs Store 2 | 0.835 |

Table S5. Correlation analysis within bacterial communities of *Chlorophytum* *comosum* and *Musa acuminata* based on 16S rRNA gene amplicon sequencing.

| Plant species | Taxon1 | Taxon2 | Correlation* | P.value |
| --- | --- | --- | --- | --- |
| *C. comosum* | *Acinetobacter* | *Pseudarthrobacter* | 0.575 | 0.008 |
|  | *Acinetobacter* | *Bacillus* | 0.595 | 0.006 |
|  | *Acinetobacter* | *Exiguobacterium* | 0.749 | <0.001 |
|  | *Pseudomonas* | *Pantoea* | 0.549 | 0.012 |
|  | *Pseudomonas* | *Brevibacillus* | 0.655 | 0.002 |
| *M. acuminata* | *Acinetobacter* | *Curtobacterium* | 0.566 | 0.022 |
|  | *Pseudomonas* | *Bacillus* | 0.605 | 0.045 |

*****Spearman's rank correlation rho. Positive value indicates that the two taxa have positive correlation.

**Table S6. Detailed completeness and contamination values and taxonomic classification of bacterial MAGs.**

| MAG ID | Host origin | Completeness | Contamination | Classification |
| --- | --- | --- | --- | --- |
| MA_MAG1 | *Musa acuminata* | 96.8 | 3.97 | *Proteobacteria;Alphaproteobacteria;Sphingomonadales;Sphingomonadaceae;Sphingomonas* |
| MA_MAG2 | *Musa acuminata* | 93.6 | 2.01 | *Proteobacteria;Alphaproteobacteria;Sphingomonadales;Sphingomonadaceae;Sphingomonas* |
| MA_MAG3 | *Musa acuminata* | 71.5 | 1.68 | *Proteobacteria;Gammaproteobacteria;Burkholderiales;Burkholderiaceae;Herbaspirillum* |
| MA_MAG4 | *Musa acuminata* | 54.8 | 0.98 | *Proteobacteria;Gammaproteobacteria;Xanthomonadales;Xanthomonadaceae;Xanthomonas* |
| CC_MAG1 | *Chlorophytum comosum* | 98.1 | 9 | *Proteobacteria;Gammaproteobacteria;Pseudomonadales;Moraxellaceae;Alkanindiges* |
| CC_MAG2 | *Chlorophytum comosum* | 86.7 | 8.51 | *Proteobacteria;Gammaproteobacteria;Pseudomonadales;Moraxellaceae;Alkanindiges* |
| CC_MAG3 | *Chlorophytum comosum* | 70.4 | 4.17 | *Proteobacteria;Gammaproteobacteria;Pseudomonadales;Moraxellaceae;Alkanindiges* |
| CC_MAG4 | *Chlorophytum comosum* | 75.4 | 3.32 | *Actinobacteriota;Actinomycetia;Propionibacteriales;Nocardioidaceae;Aeromicrobium* |
| CC_MAG5 | *Chlorophytum comosum* | 56.6 | 2.24 | *Firmicutes;Bacilli;Bacillales;Marinococcaceae;Sinobaca* |
| CC_MAG6 | *Chlorophytum comosum* | 86.5 | 8.85 | *Proteobacteria;Gammaproteobacteria;Enterobacterales;Enterobacteriaceae* |
| CC_MAG7 | *Chlorophytum comosum* | 87.8 | 1.59 | *Proteobacteria;Gammaproteobacteria;Enterobacterales;Enterobacteriaceae;Moranella* |
| CC_MAG8 | *Chlorophytum comosum* | 63.2 | 6.81 | *Actinobacteriota;Actinomycetia;Propionibacteriales;Nocardioidaceae;Aeromicrobium* |
| CC_MAG9 | *Chlorophytum comosum* | 76.0 | 0 | *Proteobacteria;Gammaproteobacteria;Enterobacterales;Enterobacteriaceae;Mikella* |
| CC_MAG10 | *Chlorophytum comosum* | 74.8 | 0 | *Proteobacteria;Gammaproteobacteria;Enterobacterales;Enterobacteriaceae;Mikella* |
